# Dihydronicotinamide riboside promotes cell-specific cytotoxicity by tipping the balance between metabolic regulation and oxidative stress

**DOI:** 10.1371/journal.pone.0242174

**Published:** 2020-11-09

**Authors:** Manoj Sonavane, Faisal Hayat, Mikhail Makarov, Marie E. Migaud, Natalie R. Gassman

**Affiliations:** 1 Department of Physiology and Cell Biology, University of South Alabama College of Medicine, Mobile, AL, United States of America; 2 University of South Alabama Mitchell Cancer Institute, Mobile, Alabama, United States of America; 3 Department of Pharmacology, University of South Alabama College of Medicine, Mobile, AL, United States of America; National Institutes of Health, UNITED STATES

## Abstract

Nicotinamide adenine dinucleotide (NAD^+^), the essential cofactor derived from vitamin B3, is both a coenzyme in redox enzymatic processes and substrate in non-redox events; processes that are intimately implicated in all essential bioenergetics. A decrease in intracellular NAD^+^ levels is known to cause multiple metabolic complications and age-related disorders. One NAD^+^ precursor is dihydronicotinamide riboside (NRH), which increases NAD^+^ levels more potently in both cultured cells and mice than current supplementation strategies with nicotinamide riboside (NR), nicotinamide mononucleotide (NMN) or vitamin B3 (nicotinamide and niacin). However, the consequences of extreme boosts in NAD^+^ levels are not fully understood. Here, we demonstrate the cell-specific effects of acute NRH exposure in mammalian cells. Hepatocellular carcinoma (HepG3) cells show dose-dependent cytotoxicity when supplemented with 100–1000 μM NRH. Cytotoxicity was not observed in human embryonic kidney (HEK293T) cells over the same dose range of NRH. PUMA and BAX mediate the cell-specific cytotoxicity of NRH in HepG3. When supplementing HepG3 with 100 μM NRH, a significant increase in ROS was observed concurrent with changes in the NAD(P)H and GSH/GSSG pools. NRH altered mitochondrial membrane potential, increased mitochondrial superoxide formation, and induced mitochondrial DNA damage in those cells. NRH also caused metabolic dysregulation, altering mitochondrial respiration. Altogether, we demonstrated the detrimental consequences of an extreme boost of the total NAD (NAD^+^ + NADH) pool through NRH supplementation in HepG3. The cell-specific effects are likely mediated through the different metabolic fate of NRH in these cells, which warrants further study in other systemic models.

## Introduction

Nicotinamide adenine dinucleotide (NAD^+^) and its phosphorylated and reduced forms (referred to as NAD(P)(H)) are functional cofactors and redox partners that participate in more enzymatic reactions than any other known vitamin-derived molecules and is intimately implicated in essential bioenergetics, anabolic and catabolic pathways [1, 2]. The NAD(H)-dependent redox processes play critical roles in cellular homeostasis and growth through their involvement in ATP generation through mitochondrial oxidative phosphorylation and glycolysis [3]. Moreover, on its own, NAD^+^ also has an important function in cellular signaling by acting as a co-substrate for post-translation modifying enzymes (Sirtuins and ARTs) and ligands for extra- and intracellular receptors and ion channels [4]. By acting as reducers of riboflavin-derived cofactors, NADH and NADPH contribute to the production of reactive oxygen species (ROS) [5]. However, NADPH is also the reducing species necessary to maintain glutathione (GSH) levels and enable ROS inhibition [6].

Accumulating evidence has suggested that the pyridinyl nucleotide pools, NAD (including NAD^+^ and NADH) and NADP (including NADP^+^ and NADPH) are the fundamental mediators of conflicting biological processes that include maintenance of the antioxidant balance and generation of ROS, which can lead to oxidative stress and cell death [7]. However, redox processes do not affect the total cellular NAD levels. Loss of the dinucleotide pool occurs through NAD^+^ consumption in NAD^+^-dependent signaling processes. The associated enzymatic processes release nicotinamide from the nucleotide framework. To maintain the NAD^+^ pool, and conversely the NADH pool, nicotinamide is readily recycled to NAD^+^. As the NAD^+^ pool is turned over several times per day in various tissues and cell types [8], an adequate supply of both NAD^+^ precursors, including nicotinamide, and NAD^+^ biosynthetic enzymes are essential for cell vitality. Sustained sub-optimal intracellular NAD^+^ levels have been shown to have long-term physiological consequences, while depletion of NAD through nutritional deficiency leads to pellagra, a debilitating and deadly disease still endemic in some regions of the world [9, 10]. Newer biomedical rationales have driven attention to NAD^+^ biosynthesis, as NAD homeostasis depends on age [11]. Studies have demonstrated that NAD^+^ levels decline with age in various tissues of rodents and humans [12]. Critically, the decrease in intracellular NAD^+^ levels is also known to cause multiple metabolic complications and age-related diseases, such as obesity, diabetes, and Alzheimer’s disease [13–15]. Supplementation of NAD^+^ precursors such as nicotinamide mononucleotide (NMN), nicotinamide riboside (NR), nicotinic acid (NA), and nicotinamide (NAM) can replete the NAD^+^ pool. Yet, not all supplements share the same efficacy in mitigating metabolic complications including insulin sensitivity, fatty liver [16] and kidney damage [17], and preventing mitochondrial disease [18, 19], Alzheimer’s disease [20, 21], cardiovascular disorders [22], and even cancer [23].

To achieve protective effects of disease states, animal models require high doses (250–1000 mg/kg/day or 1–4 mmol/kg) of NAD^+^ precursors [11, 24, 25]. These dose ranges correspond to a non-sustainable 18.5–75 g/day supplementation for an adult, limiting the ability to pursue protective effects in humans based on the animal model work. Dose optimization for NAD^+^ precursors is needed for human use and requires a better understanding of the optimal NAD^+^ levels in human tissues. Further, the efficacy and duration with which a precursor boosts NAD^+^ levels in these tissues also needs to be understood.

**Fig 1:**
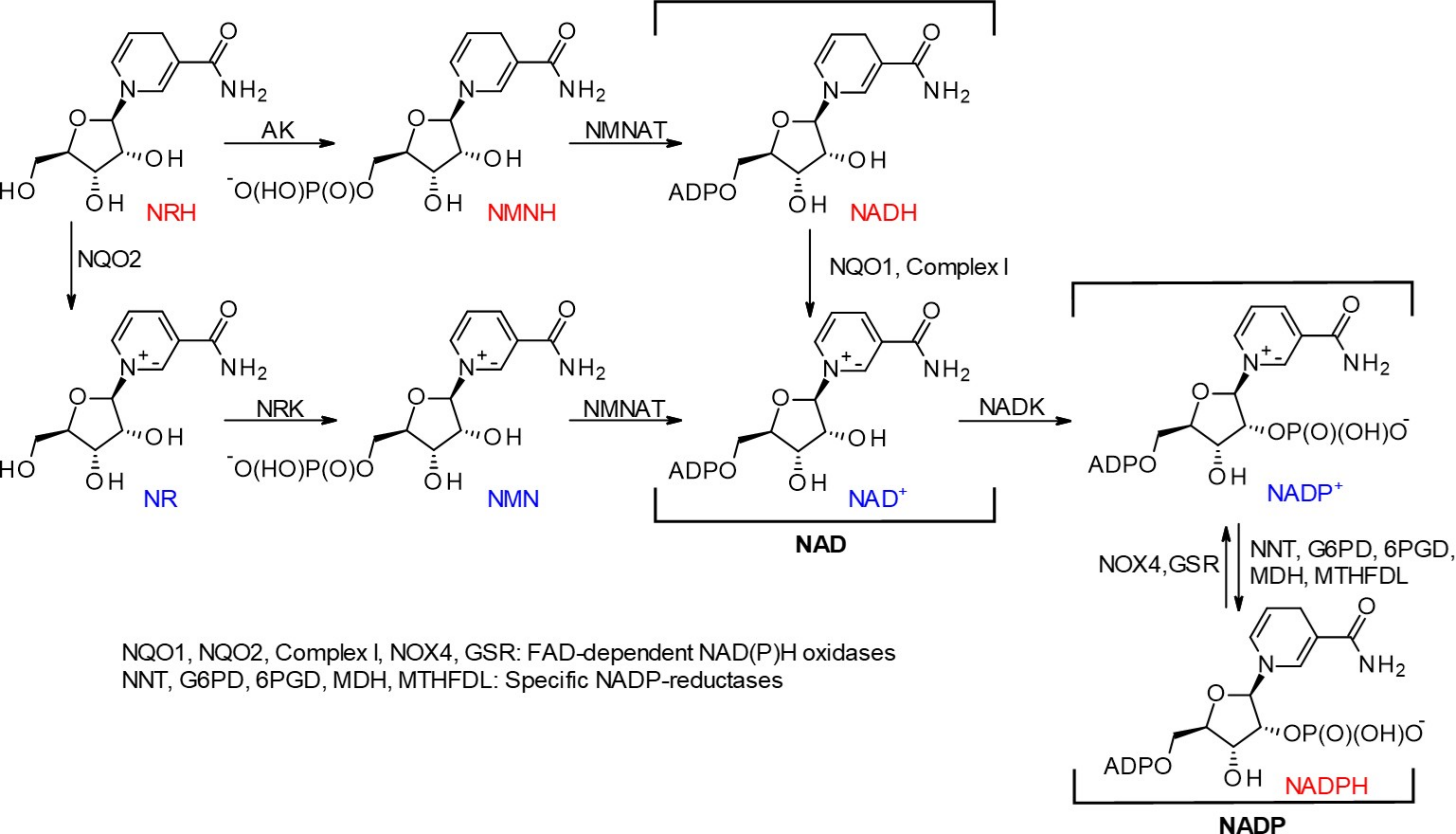
Chemistry of dihydronicotinamide riboside.

Dihydronicotinamide riboside (NRH), the reduced form of NR, a recently characterized NAD precursor [26, 27], increases NAD levels. Metabolic studies established NRH as a natural NAD precursor with endogenous NRH found in the liver [28]. NRH is a substrate for the detoxifying riboflavin-dependent enzyme NRH:quinone oxidoreductase 2 (NQO2), which converts quinones to dihydroquinones with the concomitant production of NR [29, 30]. Despite the minor chemical differences with the other NAD^+^ precursors, NRH reveals another biosynthetic pathway to NAD production, allowing further flexibility in the maintenance of intracellular NAD levels. Unlike NR, which gets phosphorylated to NMN by NR kinase, NRH is phosphorylated by adenosine kinase (AK) and is converted to the reduced form of NMN (NMNH) and subsequently to NADH and NAD^+^ [28, 31] (Fig 1). However, the low turn-over of the initial phosphorylation process means NQO2 could contribute significantly to NAD^+^ production via NR and NR kinase [28]. Recent investigations have demonstrated that NRH increased NAD^+^ levels more significantly in both cultured cells and mouse models than current supplementation strategies with NR or vitamin B3 (nicotinamide and niacin) [31, 32]. Yet, the consequences of the substantial increase in the total intracellular NAD pools fueled by NADH, instead of NAD^+^, are still unknown. These consequences may be critical to cell health since NADH is not a substrate for sirtuins or ARTs. NADH can only contribute to sirtuin or ART regulatory processes once it is oxidized to NAD^+^ with the concomitant reduction of endogenous species (*e*.*g*., O_2_, pyruvate, etc.) [3, 33]. Since the consequences of extreme boosts in NAD levels are not fully understood, caution in the use of NRH must be exercised as it may have adverse effects. In this study, we describe our investigations to show the cell-specific effects of acute NRH exposure in mammalian cells (S1 Graphical abstract).

## Materials and methods

### Cell culture

Human Embryonic Kidney containing the SV40 T-antigen (HEK293T) and the human hepatocellular carcinoma cell line (HepG3) were purchased from ATCC (Manassas, VA). The cells were grown at 37°C in a 5% CO_2_ incubator in Dulbecco’s modified Eagle’s medium (DMEM, Hyclone, Logan, UT, USA; 4.5 g/L glucose and L-glutamine) supplemented with 10% fetal bovine serum (FBS, Atlanta Biologicals, Flowery Branch, GA), and 1% sodium pyruvate (Gibco, Carlsbad, CA). Cell passages between 4 and 15 were used for all reported experiments. Cells were routinely tested for mycoplasma using the Lonza MycoAlert kit (Walkersville, MD) and found to be free of mycoplasma contamination.

### Residual NRH in culture media measured by ^1^H NMR

The supernatants were examined at 0, 1, and 24 h by ^1^H NMR analysis for the presence of any remaining NRH after supplementation at 100 μM in HEK293T and HepG3. Samples were prepared as follows: 450 μL of cell culture supernatant was mixed with 50 μL of D_2_O, and the resulting mixture was vortexed three times. NMR spectral acquisition (ns = 2048) was then performed using a Bruker Avance III HD NMR spectrometer equipped with 400 MHz magnet Ultrashield Plus, with temperature fixed to 300 K for all NMR measurements. TopSpin 3.2 (Bruker BioSpin) was used for all NMR spectral acquisition and preprocessing, and the automation of sample submission was performed using ICON-NMR (Bruker BioSpin). All samples were automatically shimmed. The FID were processed automatically using ICON-NMR (Bruker BioSpin), and phasing was refined manually. NRH, NR, and nicotinamide were all detected (S1 Fig). NRH is oxidized to NR in aqueous solution over time, while nicotinamide is present at ~16 μM in DMEM. Nicotinamide accumulates in the media over time as NRH is consumed by the cells.

### Cytotoxicity studies

Cytotoxicity was determined by CellTiter-Fluor^TM^ Cell Viability assays (Promega Corporation, Madison, WI). HEK293T and HepG3 cells were seeded at a density of 5 X 10^3^ cells/well and 1 X 10^4^ cells/well respectively in a 96-well clear bottom black plate and incubated overnight (ON) at 37 °C in a 5% CO_2_ incubator. For HEK293T cells, poly D-lysine coated black plate was used to avoid washing off the cells during medium replacement. The following day, cells were exposed to 100, 250, 500, 750, or 1000 μM concentrations of dihydronicotinamide riboside (NRH) [26] for 24 h. For the Mito-TEMPO co-exposure study, 25 and 50 nM of Mito-TEMPO (Enzo Life Sciences, Inc., Farmingdale, NY) was co-exposed with 100 and 500 μM of NRH for 24 h. NRH was dissolved in Dulbecco’s phosphate-buffered saline (PBS, Hyclone, Logan, UT) at 10 mM then diluted to the final working concentrations in the medium. Mito-TEMPO was dissolved in DMSO and further diluted into the media to obtain the desired concentrations. After 24 h exposure, medium containing NRH and Mito-TEMPO was replaced with fresh medium, and cells were further incubated for another 48 h (total 72 h) at 37 °C in a 5% CO_2_ incubator. At the end of the incubation period, 100 μl of 2X CellTiter-Fluor^TM^ Viability reagent was added in each well containing 100 μl of medium, mix briefly, and plates were incubated for another 30 min at 37°C in a 5% CO_2_ incubator. Measure resulting fluorescence intensity in a microplate reader (Infinite® M1000 PRO, TECAN, Männedorf, Switzerland) with a fluorimeter at excitation/emission (Ex/Em) of 380/505 nm. Cells (triplicate wells for each chemical concentration) were performed in at least three biological repeats, and results were expressed as the number of cells in drug-treated wells relative to cells in control wells (% Viability) ± standard error of the mean (SEM).

### Immunoblotting

The cell death mechanism of NRH induced cytotoxicity was examined by assessing the apoptotic markers using immunoblotting. Briefly, HEK293T and HepG3 cells were cultured in 100 mm and 65 mm petri dish respectively at a density of 1 X 10^6^ cells per dish and incubated ON at 37°C in a 5% CO_2_ incubator. For apoptotic markers and other protein detection, cells were untreated, treated with 500 μM NRH for 24 h, and cell pellets were collected after 24, 48, and 72 h for immunoblotting. For 24 h samples, cell pellets were collected immediately at the end of the exposure period. For 48 and 72 h samples, medium containing NRH was removed and replaced with fresh medium, and cell pellets were collected at the desired exposure period and stored at -80°C. As for NQO2, cells were treated with 100 μM NRH for 1, 4, and 24 h. Pellets were lysed in lysis buffer (25 mM β-glycerolphosphate, 50 mM Tris-HCl, pH 7.5, 150 mM NaCl, 0.2%Triton X-100, and 0.3% NP-40) along with 1X Halt protease and phosphatase inhibitor (Pierce, Waltham, MA), and incubated on ice for 30 min. Lysates were then centrifuged at 12,000 rpm for 15 min at 4°C, and the supernatant fraction containing protein was retained. Protein concentrations were determined by Bradford Quick Start protein assay (Bio-Rad, Hercules, CA). A 30 μg of protein sample was separated on 4−15% SDS-PAGE and transferred onto nitrocellulose membranes (Bio-Rad). Membranes were blocked in 5% skim milk in Tris-buffered saline (TBS, VWR Life Sciences) containing 0.1% Tween 20 (TBST) and then incubated with the following primary antibodies: BAX (1:300, N-20: sc-493) and NQO2 (1:500, sc-271665) from Santa Cruz Biotechnology, Inc. (Dallas, TX); NQO1 (1:5000, ab-80588) from abcam (Cambridge, MA); PUMA (1:1000, D30C10) from Cell Signaling Technology, Inc. (Danvers, MA); and NOX4 (1:1000, MABC616) and α-tubulin (1:5000, T9026) from Millipore Sigma (St. Louis, MO).

### Cellular ROS measurements

Cellular reactive oxygen species (ROS) was measured by the chemically reduced form of cell-permanent 2’,7’-dichlorodihydrofluorescein diacetate (CM-H_2_DCFDA) fluorescein indicator (Life Technologies Corporation, Grand Island, NY). HEK293T and HepG3 cells were seeded at a density of 1 X 10^4^ cells/well and 2.5 X 10^4^ cells/well respectively in a 96-well clear bottom black plate and incubated ON at 37°C in a 5% CO_2_ incubator. For HEK293T cells, poly D-lysine coated black plate was used to avoid washing off the cells. The next day, cells were treated with no chemical (control), 100 μM NRH for 1, 4, and 24 h and 250 μM tert-butyl hydrogen peroxide (TBHP) for 1 h, as a positive control for ROS production. At the end of the exposure period, cells were washed in PBS and further allowed to react with 2.5 μM of CM-H_2_DCFDA (a stock of 1 mM of CM-H_2_DCFDA was prepared in anhydrous DMSO (Sigma-Aldrich) and further diluted to 2.5 μM in PBS) for 15 min at 37°C in a 5% CO_2_ incubator. Fluorescence intensity of the samples was measured at Ex/Em of 495/525 nm using Infinite® M1000 PRO, TECAN microplate reader. The number of cells was normalized by adding 1:1000 diluted Hoechst 33342 (Life Technologies) stain to the sample wells for 10 min and further measuring fluorescence at Ex/Em 355/460 nm. The obtained values for the treated samples were compared relative to the control values, and the fluorescence intensity was expressed as the average of the three biological replicates that were performed on different days ± SEM.

### Intracellular NAD(P)H measurements

NAD(P)H levels were measured using the endogenous autofluorescence of these dinucleotides when they are excited with a 355 nm laser, as described in [34]. Briefly, HEK293T and HepG3 cells were seeded in an 8-well chambered coverglass at a density of 2 X 10^4^ cells/chamber and 4 X 10^4^ cells/chamber, respectively and incubated ON at 37°C in a 5% CO_2_ incubator. The next day, cells were treated with medium only and 100 μM NRH for 1, 4, and 24 h. After indicated treatment time, medium containing treatment was removed, and cells were washed twice with medium and fresh medium was added to image cells using a Nikon ARsi scanning confocal microscope modified to include a 355 nm laser (PicoQuant, Berlin, Germany) as previously described in Holton et al. [35]. NAD(P)H fluorescence imaging was performed using a UV passing 40X C-Apochromat (numerical aperture (NA) 1.2) oil immersion objective, and images were collected with an iXON III EMCCD camera (Andor Technology, Belfast, UK). A region of interest within a selected field of cells was scanned with the 355 nm laser beam while collecting a time-lapse series at the maximum frame rate of the camera for 2 min. Emitted light was filtered with a UV-2E filter cube (Nikon Instruments). A composite image was generated using maximum intensity projection over the time-lapse series and then thresholded to create a binary mask defining the NAD(P)H signal. Intensity values within the mask were measured for least 50 ± 15 cells using NIS-Elements software, and the values were reported as binary mean intensity relative to control values. Statistical analysis was performed using one-way ANOVA followed by Dunnett's multiple comparison test.

### Mass spectrometry analysis

HEK293T and HepG3 cells were seeded at a density of 2.5 X 10^6^ cells/well and 5 X 10^6^ cells/dish respectively in a 100 mm petri dish and incubated ON at 37°C in a 5% CO_2_ incubator. The next day, cells were treated with no chemical (control), 100 μM NRH for 1, 4, and 24 h. At the end of the exposure period, media was aspirated, cells were scrapped, and pellets were resuspended in PBS for cell counting. Equal number of cell pellets were collected in 1.5 mL Eppendorf tubes and were immediately used for the extraction process without storing at -80 ^o^C. Solvents (CHCl_3_, MeOH & H_2_O) were degassed, and 200 μL of degassed MeOH was added in each Eppendorf tubes containing cell pellet and sonicated at 4°C for three times (10 sec each) to rupture the cell wall. After sonication, the sample was incubated at 0°C for 2 minutes on ice, followed by the addition of 200 μL MeOH, 400 μL CHCl_3,_ and 200 μL H_2_O and vortex for 30 seconds. The resulting mixture was centrifuged at 13,000 rpm at 4°C for 15 min. Aqueous and organic phases were separated by centrifugation and were collected separately. 400 μl of the aqueous phase was transferred in 1 mL Eppendorf tube and flash-frozen in liquid nitrogen and freeze-dried. Freeze-dried samples were reconstituted in 35μl of 10mM ammonium acetate, and 6 μl was used for each injection. LC-separation was achieved on Agilent 1200 series Liquid Chromatography (LC) with Agilent Zorbax 300SB (C18, 2.1 x 150 mm) column. The flow rate was set at 50 μl per minute initially and ramped it up to 150 μl /min in 20 min and held there until the end of the run. Mobile phase A is 10 mM ammonium acetate in 0.1% formic acid (Fluke, NY), Mobile phase B is 0.1% formic acid in methanol (Thermo Fisher Scientific, Waltham, MA). Gradient conditions were 2% Mobile phase B initially and raised to 95% in 20 min, hold it for 5 min, and brought it back to 2% in 15 min and equilibrated for 10 min. LC eluent was introduced to Thermo LTQ Orbitrap XL Mass spectrometer (Thermo Scientific, San Jose, CA, USA), equipped with Electrospray Ionization. Spray voltage was set at 3.5 kV, capillary voltage, and temperature at 50V and 275°C, respectively. Data were acquired in positive ionization in SIM mode, and the acquired data were processed in Xcalibur. Two technical replicates were run for each biological repeats. The graph was plotted as the average of the peak values relative to their respective control ± SEM of at least two biological repeats.

### Measurement of GSH/GSSG

The cell’s capacity to generate reduced glutathione (GSH) and oxidized glutathione (GSSG) was quantified using the GSH/GSSG-Glo^TM^ assay (Promega Corporation, Madison, WI). Briefly, HEK293T and HepG3 cells were cultured in a 96-well clear bottom white plate at a density of 1 X 10^4^ cells/well and 2.5 X 10^4^ cells/well, respectively, and incubated ON for cells to attach. Following day, cells were treated with medium only and 100 μM NRH for 1, 4, and 24 h. After 24 h, cells were counted on a Celigo S imaging instrument to normalize for variations in cell density. Next, medium alone or medium containing NRH was removed, and the assay was performed according to the manufacturer’s protocol. Briefly, 50 μl of total glutathione lysis reagent or oxidized glutathione lysis reagent was added per well to immediately lyse the cells by continuously shaking the plate for 5 min at room temperature. Followed by the addition of 50 μl of luciferin generation reagent per well, the plate was briefly shaken and then incubated at room temperature for 30 min. Finally, 100 μl of luciferin detection reagent was added to the above mixture and incubated at room temperature for 15 min. Following assay incubation, luminescence was measured (in relative light units, RLU) on Infinite® M1000 PRO, TECAN microplate reader. Luminescence values were normalized to cell count, and the total glutathione and GSSG were measured. The GSH/GSSG ratio was calculated by subtracting two times normalized values of GSSG from the normalized total glutathione and divided this by normalized GSSG values (as shown in Eq 1).

$$Ratio\;GSH/GSSG=\frac{\mu M\;total\;glutathione–\left(\mu M\;GSSG\;X\;2\right)}{\mu M\;GSSG}$$ (1)

### Detection of mitochondrial superoxide

Mitochondria superoxide formation was measured using the mitochondrial-targeted, superoxide-sensitive fluorogenic probe MitoSOX^TM^ Red (Life Technologies). Briefly, HEK293T and HepG3 cells were plated in an 8-well chamber dish at a density of 2 X 10^4^ cells/well and 4 X 10^4^ cells/well, respectively, and incubated overnight (ON) at 37°C in a 5% CO_2_ incubator. The next day, cells were treated with medium only and 100 μM NRH alone and in combination with 50 nM Mito-TEMPO for 1, 4, and 24 h. Following the 24 h exposure period, the medium was discarded, and cells were incubated with 1 μM MitoSOX^TM^ Red reagent (diluted in medium) for 10 min at 37°C, protected from light. The cells were washed gently two times with PBS, and warm PBS was used for imaging.

Stained cells were captured using a Nikon A1rsi scanning confocal microscope using a 20X C-Apochromat (NA 0.75) air immersion objective. MitoSOX Red stain images were acquired at a resolution of 1024x1024 resolution using the 561 nm laser. For analysis, binary masks were generated from each image, and the binary intensity of MitoSOX Red staining per image was calculated. For each treatment, at least 50 ± 15 cells were measured using NIS-Elements software, and the values were reported as fluorescence intensity of the mean binary intensity ± SEM of three biological replicates.

### Monitoring of mitochondrial membrane potential

The mitochondrial membrane potential of cells was monitored by tetramethylrhodamine, ethyl ester (TMRE) Mitochondrial Membrane Potential Assay Kit, according to manufacturer protocol (BioVision, Milpitas, CA). Briefly, HepG3 cells were seeded at a density of 2.5 X 10^4^ cells/well in a 96-well clear bottom black plate and incubated ON at 37°C in a 5% CO_2_ incubator. For HEK293T cells, 1 X 10^4^ cells/well were seeded in poly D-lysine coated black 96-well plate to avoid washing off the cells. The next day, cells were treated with medium only (control), 100 μM NRH for 1, 4, and 24 h. Carbon cyanide 4-(trifluoromethoxy) phenylhydrazone (FCCP, BioVision) at 30 μM was used as a negative control and incubated at 37°C for 20 min before dosing all samples with TMRE (200 nM) for 25 min at 37°C. Following incubation, media containing dye was discarded, and the cells were washed twice with PBS and replaced the final wash with PBS to read fluorescence with Tecan M1000 microplate reader at Ex/Em = 549/575 nm. Hoechst 33342 (Life Technologies) stain (1:1000 dilution) was used to normalize the experimental fluorescence values with cell density variation. The obtained values for the treated samples were compared relative to the control values, and the fluorescence intensity was expressed as the average of the three biological replicates ± SEM.

### Quantification of mitochondrial DNA damage

Mitochondrial DNA damage was quantified by the quantitative polymerase chain reaction (qPCR) assay, which measures DNA damage in the mitochondrial and nuclear genomes without isolation of mitochondria, as previously described [36]. In brief, HEK293T and HepG3 cells were cultured in 100 mm and 65 mm petri dish respectively at a density of 1 X 10^6^ cells per dish and incubated overnight at 37°C in a 5% CO_2_ incubator for cells to adhere. The following day, cells were untreated, treated with 100 μM NRH for 1, 4, and 24 h. After the designated incubation time, the culture medium was aspirated, and cells were scraped, and pellets were stored at -80°C ON. Genomic DNA from the whole-cell pellet was purified by Purelink® Genomic DNA Mini Kit (Life Technologies).

Recovered genomic DNA was quantified using AccuBlue^TM^ Broad Range dsDNA Quantification Kits with 9 DNA Standards, according to the manufacturer’s instructions (Biotium, Fremont, CA). DNA recovery was uniform, and 3.75 ng/μl of DNA per treatment was used to run the qPCR with MT-CO1 VIC (Hs02596864_g1) to determine the mitochondrial copy number and normalize the quantity of mitochondrial DNA for damage analysis. The normalized DNA was amplified for specific mitochondrial DNA targets with 10 μM of Mito-long PCR primer mixture (containing both forward (F) and reverse (R) primers, F: GCTTCACTCAGCCATTTTACCTCACCC and R: GGTTAATTTTGCGTATTGGGGTCATTGGT) using Elongase (Life Technologies). Finally, the amplified DNA samples are run on 1% agarose gel for 30 min at 100 V. The amplified DNA band intensity is inversely proportional to the amount of mitochondrial DNA damage and was quantified relative to control untreated DNA using the Image Lab software. The amount of mitochondrial DNA damage was expressed as the average of the three biological replicates ± SEM.

### Mitochondrial stress test assay

Mitochondrial respiratory function of HEK293T and HepG3 cells were measured with a Seahorse XF24 cellular flux analyzer (Agilent Technologies, Santa Clara, CA, USA) according to the manufacturer’s recommendations. Briefly, HEK293T and HepG3 cells were seeded in 100 μl growth medium at the density of 5 X 10^3^ cells/well and 1.3 X 10^4^ cells/well respectively and placed ON in 37°C incubator with 5% CO_2_. The next day, 150 μl growth medium was added, and cells were grown for another 24 h at 37°C incubator with 5% CO_2_. The next day, cells were either left untreated or treated with 100 μM NRH for 1 h and 24 h. Following treatment duration, wells were washed with warmed Seahorse serum-free XF medium (XF base medium supplemented with 25 mM glucose, 2 mM glutamine, and 1 mM sodium pyruvate; pH 7.4) and then filled with 0.5 ml of fresh Seahorse assay medium. Immediately culture dish was placed in the Celigo cytometer for direct cell counting and then incubated in a CO_2_-free incubator at 37°C for at least 1 h to pre-equilibrate with the assay medium before running the plate in the Seahorse machine. The Mito Stress test assays were performed by loading with pre-warmed oligomycin (Sigma), FCCP (Cayman Chemical), rotenone & antimycin A (Sigma) into injector ports A, B, and C, respectively. The final concentrations of injections were 10 μM oligomycin, 7.5 μM FCCP, 15 μM of rotenone, and antimycin A. The cartridge with loaded chemicals was incubated in CO_2_-free incubator for at least 10 min before the XF24 analyzer calibrated it. After calibration, the cartridge plate was replaced with the culture plate, and the assay was continued according to the manufacturer’s recommendation. Using Seahorse Wave controller 2.4 software (Agilent), oxygen consumption rate (OCR) and extracellular acidification rate (ECAR) were monitored under basal conditions. This was followed by the sequential addition of oligomycin, FCCP, and rotenone & antimycin A to estimate the contribution of individual parameters for basal respiration, ATP production, proton leak, maximal respiration, spare respiratory capacity, and non-mitochondrial respiration. The mito stress test report generator by the XF24 analyzer automatically calculated the XF cell mito stress test parameters from Wave after cell count normalization and was exported to Excel. For OCR curves and individual parameters, the values are presented relative to control as the mean ± SEM of three biological repeats in all experiments performed with at least 4 replicate wells in the Seahorse XF24 analyzer. The significance level was determined by performing Student’s t-test between the control and the treated samples with results considered significant when P < 0.05.

### Statistical analysis

Unless stated otherwise, data are represented as ± SEM of three biological replicates. Biological replicates are individually treated samples, whereas technical replicates are repeat assays of the same biological replicate. Comparisons between groups were performed by one-way analysis of variance (ANOVA) and Student’s t-test to establish reported p-values. All statistical analyses were performed using Prism 7 (GraphPad Software, Inc).

## Results

### NRH induces apoptosis-mediated cytotoxicity in HepG3 but not in HEK293T at 24 h

**Fig 2:**
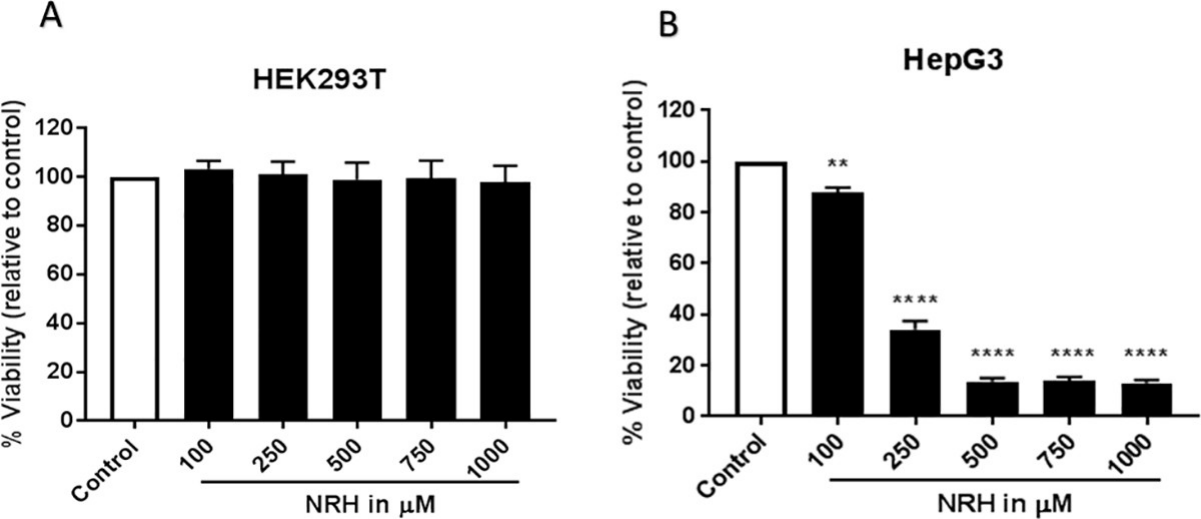
NRH treatment dose-dependently decreased the number of surviving HepG3 cells. HEK293T (A) and HepG3 (B) cells were exposed to 100, 250, 500, 750 and 1000 μM of NRH for 24 h. After 24 h, NRH was removed and replaced with fresh medium, and cells were allowed to grow for another 48 h before performing CellTiter-Fluor^TM^ Viability assay. Results are expressed as the mean fluorescence intensity to that of the control (% Viability) ± SEM. Statistical significance: * P < 0.05, *** P < 0.001.

To determine the cytotoxicity of NRH in HEK293T and HepG3 cells, we treated cells with increasing doses of NRH (100–1000 μM) for 24 h, then assessed cell viability the Cell Titer Fluor assay (Fig 2). The 24 h treatment with NRH significantly decreased the survival of the HepG3 cells by 15–90%, with slight cytotoxicity (15 ± 2.1%), moderate cytotoxicity (50 ± 3.3%), and highest cytotoxicity (90 ± 1.4%) observed when treated with 100, 250 and ≥ 500 μM NRH concentrations, respectively (Fig 2B). In contrast, cytotoxicity was not observed in HEK293T cells over the same dose range of NRH (Fig 2A).

**Fig 3:**
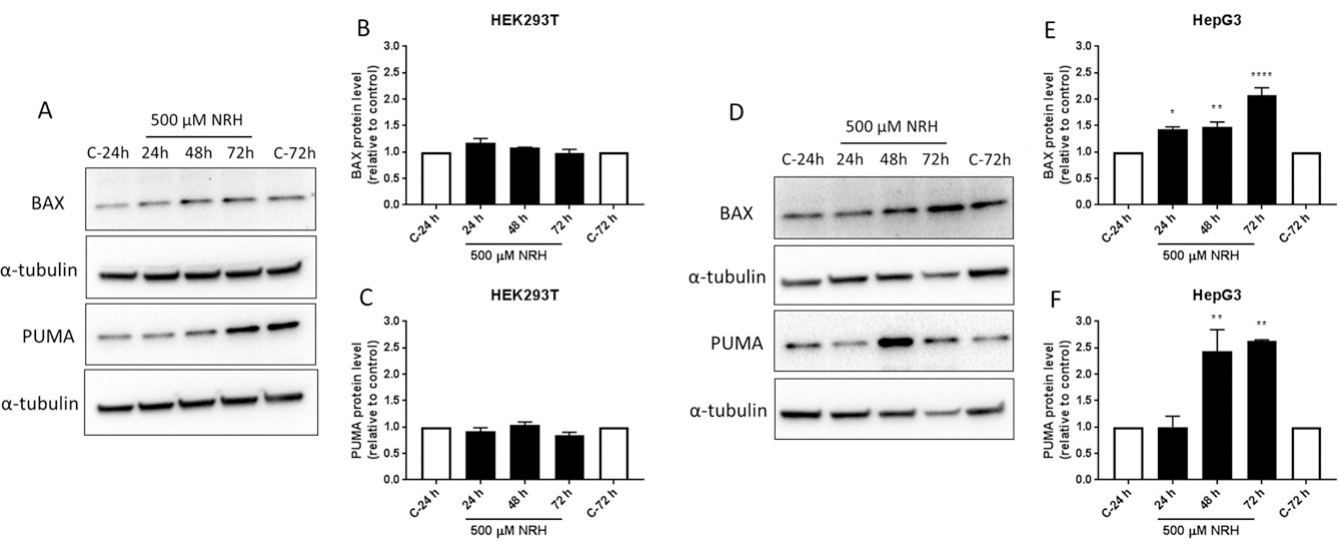
NRH induced PUMA and BAX-mediated apoptosis in HepG3 cells. Apoptosis in HEK293T and HepG3 cells were assessed using immunoblot after 24, 48, and 72 h of NRH exposure. Non-significant increases in the BAX and PUMA protein expression levels were observed in HEK293T cells (A). However, significantly increased expression of the apoptotic markers, BAX and PUMA were observed in HepG3 (D). The graph shows the protein expression levels relative to controls in HEK293T (B and C) and in HepG3 (E and F) cells. Results are expressed as the average of at least two biological replicates ± SEM. Statistical significance: * P < 0.05, ** P < 0.01, **** P < 0.001.

To examine the mechanism by which NRH exposure induced cell death, HEK293T and HepG3 cells were exposed to NRH at IC_90_ concentration (*i*.*e*., 500 μM) for 24 h, then assessed the protein expression levels of the apoptotic markers after 24, 48, and 72 h using immunoblot (Fig 3). HEK293T cells exposed to NRH showed no significant change in the apoptotic markers, BAX or the p53 upregulated modulator of apoptosis (PUMA), at all the time points (Fig 3A–3C). As expected, HepG3 cells showed a significant increase in BAX levels at 24 h (40 ± 3.5% increase, relative to control), which further increases at 48 h (50 ± 8.6%) and attained maximum expression at 72 h (110 ± 13.8%) (Fig 3D and 3E). We further demonstrated that this BAX-induced apoptosis is mediated by PUMA, which showed significantly increased expression levels at 48 h (140 ± 41.2%) and 72 h (150 ± 2.5%) of NRH exposure (Fig 3D and 3F).

### NRH induces oxidative stress at supplementation levels

The IC_90_ concentration of 500 μM far exceeds supplementation doses in humans. A far more physiologically relevant supplementation dose is 100 μM, which still showed 15 ± 2.1% cytotoxicity. We selected the 100 μM concentration for further study due to its physiological relevance and published work with NR and NRH, showing the efficient restoration of the intracellular NAD pools and cells' metabolic activity with that concentration [37]. At 100 μM, NRH is reported to increase neuronal NAD^+^ levels by approximately 5-fold over control [32]; however, in the present study, NRH is exposed to HepG3 at this concentration resulted in 15 ± 2.1% cytotoxicity (Fig 2B). Thus, we examined the mechanisms by which 100 μM NRH caused this slight cytotoxicity. Since oxidative stress is one of the key inducers of cell death under many circumstances [38], we tested the hypothesis that NRH induced cell-specific cytotoxicity is mediated by the generation of cellular reactive oxygen species (ROS). To evaluate NRH induced cytotoxic ROS, we exposed HEK293T and HepG3 cells to 100 μM NRH for 1, 4, and 24 h and examined changes in the intracellular ROS levels using the fluorescent reporter, CM-H_2_DCFDA.

**Fig 4:**
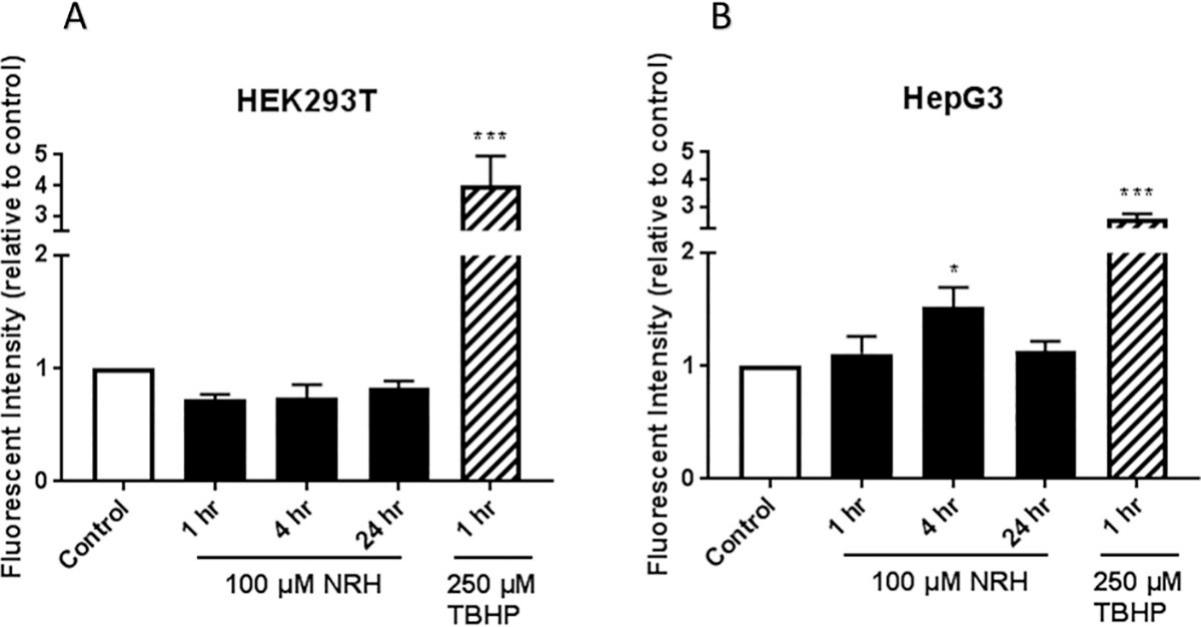
NRH exposure increased cellular reactive oxygen species (ROS) in HepG3 cells. ROS levels in HEK293T (A) and HepG3 (B) cells were assessed after 1, 4, and 24 h of NRH exposure using CM-H_2_DCFDA. Tert-butyl hydrogen peroxide (TBHP) at 250 μM exposure for 1 h was used as a positive control. Results are expressed as fluorescence intensity of treated samples relative to control of the average of the three biological replicates ± SEM. Statistical significance: * P < 0.05, *** P < 0.001.

No increase in cellular ROS was observed in HEK293T cells exposed to 100 μM NRH for the 1, 4, or 24 h (Fig 4A). In HepG3 cells, NRH induced an increase in ROS levels within 1 h that became more significantly substantial after 4 h of exposure. By 24 h, the ROS levels within the cell had returned to the levels observed at the 1 h NRH exposure (Fig 4B). The cell-specific increase in ROS seen in HepG3 suggests a different cellular fate for NRH in these cells.

### NRH exposure alters NAD(P)(H) and glutathione levels

NRH is readily intracellularized as the levels of NRH measured by ^1^H NMR in the cell supernatants (S1 Fig). Once intracellularized, NRH is converted to NADH via adenosine kinase [28, 31] and to NAD^+^ via NQO2 and NR kinase. To evaluate whether an increase in intracellular NRH increases the NAD(P)H pools and produces an NAD^+^/NADH and NADP^+^/NADPH imbalance linked to excessive ROS accumulation, oxidative stress, and the slight cytotoxicity observed, we exploited the intrinsic autofluorescence of NADH/NADPH when excited by 355 nm light [39]. NRH possesses this intrinsic autofluorescence and could contribute to the reading. However, while the rate and levels at which NRH is taken up by the cells is unknown, it is likely that its contribution rapidly declines as NRH is converted to NAD^+^ and NADH in these cells. Therefore, we consider the autofluorescent signal as primarily generated by the NAD(P)H species within the cell.

**Fig 5:**
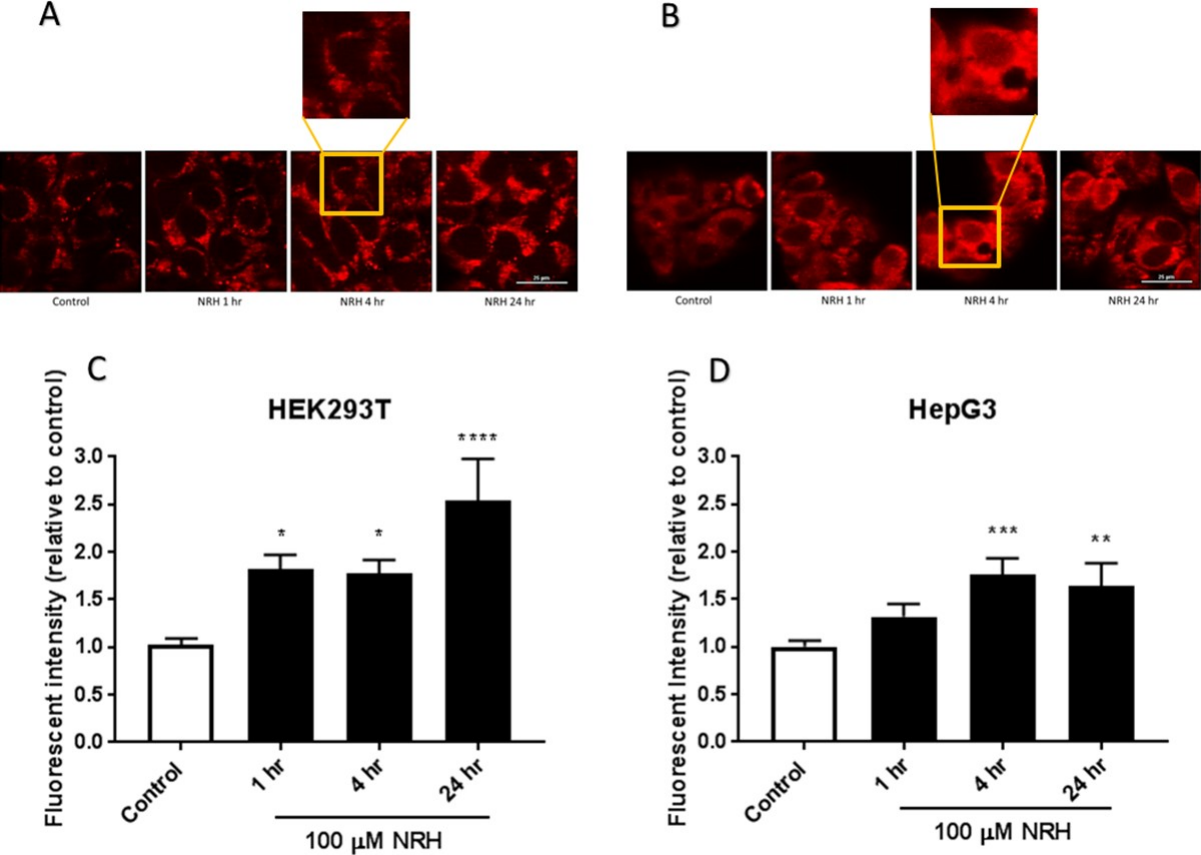
NRH exposure increased NADH/NADPH levels in HEK293T and HepG3. Autofluorescent images of endogenous NAD(P)H in untreated and 100 μM NRH exposed (A) HEK293T and (B) HepG3 cells. Quantification of fluorescent signal for untreated and 100 μM NRH treated (C) HEK293T, and (D) HepG3 cells. Results are expressed as the mean intensity of the fluorescent values relative to the control ± SEM calculated from three biological replicates. Statistically significance: * P < 0.05, ** P < 0.01, *** P < 0.005 and **** P < 0.001. Representative images are shown in part A and B from one of the three replicates, scale bars: 25 μm.

The cellular distribution of NAD(P)H in both cell lines is mostly cytoplasmic and mitochondrial before NRH treatment (Fig 5). The HEK293T cells showed an increase in NAD(P)H levels after 1 and 4 h of NRH exposure, with a more significant increase in the fluorescence response after 24 h of NRH exposure (Fig 5C). After NRH exposure, the autofluorescence is observed more intensely within the mitochondria of HEK293T cells (Fig 5A, insert). The HepG3 cells showed a significant increase in autofluorescence after 4 h with a slight decrease after 24 h exposure (Fig 4D). The autofluorescence is mostly observed within the mitochondria of the HepG3 cells, but there is also an increase in signal within the nuclear compartment (Fig 5B, insert).

**Fig 6:**
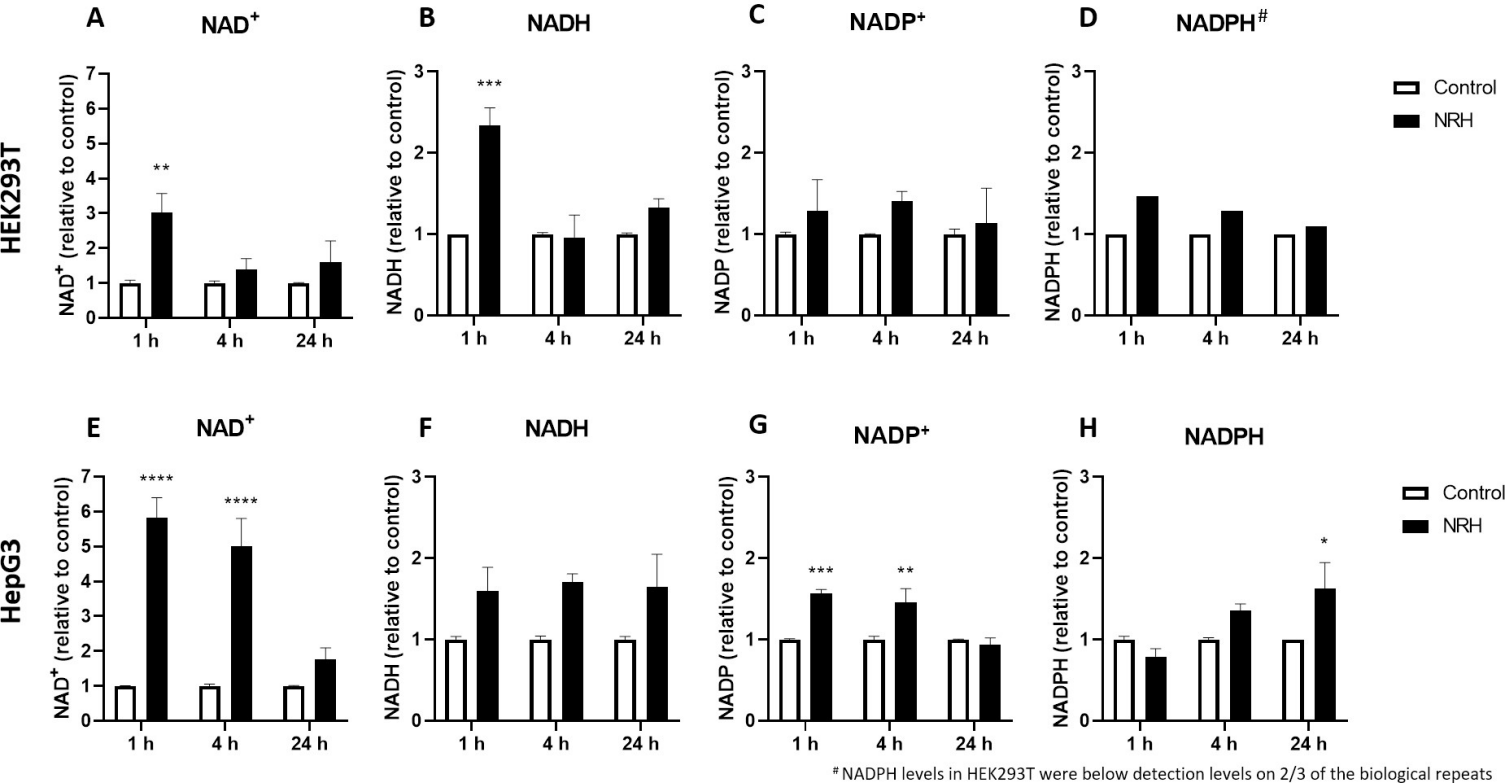
NRH exposure altered NAD(H) and NADP(H) in HEK293T and HepG3 cells. Measurements of (A) NAD^+^, (B) NADH, (C) NADP^+^ and (D) NADPH in HEK293T cells; (E) NAD^+^, (F) NADH, (G) NADP^+^ and (H) NADPH in HepG3 cells exposed to media (control) and 100 μM NRH for 1, 4, and 24 h. Results are expressed as the average peak values relative to control ± SEM calculated from three biological replicates. Statistical significance: * P < 0.05, ** P < 0.01, *** P < 0.005 and **** P < 0.001.

We performed mass spectrometry (MS) analysis to differentiate between NADH and NADPH changes in the whole cell, using relative quantification to show the more dynamic behavior [40]. We observed a gradual increase in NAD^+^ (3 fold) and NADH (2.3 fold) levels in HEK293T at 1 h, which then returned to levels comparable to control after 4 and 24 h of NRH exposure (Fig 6A and 6B). Critically, the NADP^+^ levels do not appear to change significantly in HEK293T (Fig 6C), whereas NADPH, barely detectable, showed little change following NRH exposure (Fig 6D). Strikingly, the NAD+ levels in HepG3 cells increased by more than 5 fold within the first 4 h of NRH exposure, before returning to a level similar to that of control over time (Fig 6E). NADH, NADP^+,^ and NADPH also appear to benefit from this NAD^+^ burst in HepG3 cells after NRH exposure. Although non-significant, NADH levels increased by more than 1.5 fold in HepG3 cells at 1, 4, and 24 h of NRH exposure (Fig 6F). As for the NADP^+^ pool, significantly increased levels were observed at 1 and 4 h for NADP^+^ and NADPH at 24 h in HepG3 cells (Fig 6G and 6H). This observation can be accounted for by NADPH production from NADP^+^ and NADH's concomitant oxidation via nicotinamide nucleotide transhydrogenase or other enzymes. Notably, the NADPH levels in HepG3 appear to drop following 1 h incubation but are restored substantially upon extended exposure. Overall, NRH led to a greater change in the total levels of NAD(P)(H) pools in HepG3, compared to HEK293T and an early burst of NAD^+^ in HepG3 rather than of NADH in HEK293T (NAD^+^/NADH >3.5 (HepG3) vs. NAD^+^/NADH <1.5 (HEK293T)) within the first hour of exposure.

**Fig 7:**
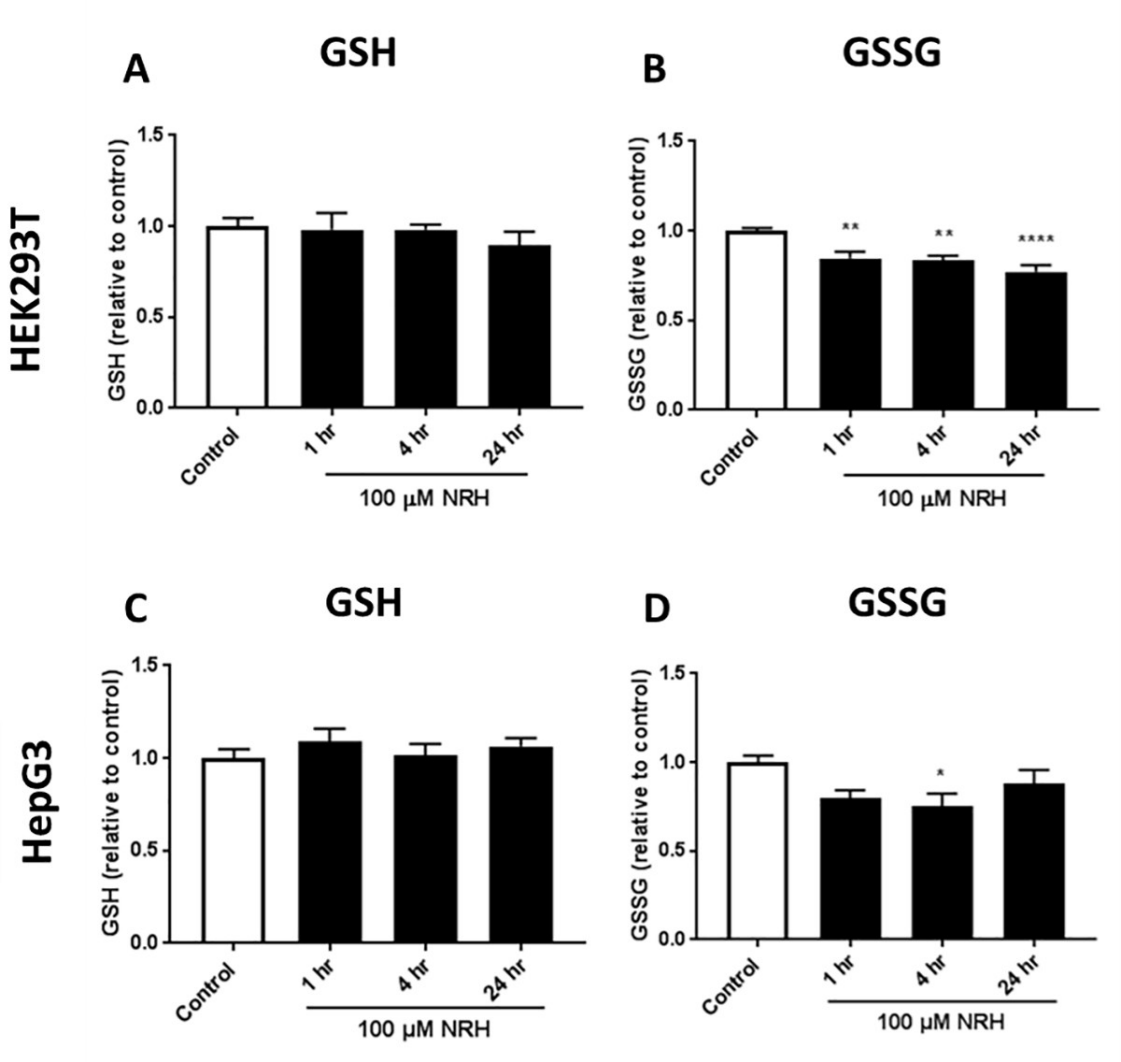
NRH exposure altered glutathione redox in HEK293T and HepG3 cells. Measurements of: (A) GSH, and (B) GSSG in HEK293T cells; (C) GSH, and (D) GSSG in HepG3 cells treated with NRH. Results are expressed as the mean luminescence values relative to the control ± SEM calculated from three biological replicates. Statistical significance: * P < 0.05.

Given these changes in NADP(H), we assessed changes in reduced and oxidized glutathione (GSH/GSSG) to examine changes in antioxidant behavior, since the maintenance of GSH levels is exclusively accomplished by the contribution of NADPH. Total intracellular GSH and GSSG were examined 1, 4, and 24 h after exposure to 100 μM NRH (Fig 7). In HEK293T cells, a significant decrease in GSSG was observed after NRH exposure, with no significant change observed in GSH levels (Fig 7A and 7B). As the NRH exposure duration increases, GSSG levels continue to decrease with 24 h showing the highest effect of NRH in HEK293T cells (Fig 7B). This is consistent with a feedback inhibition of GSH’s de novo biosynthesis. The HepG3 cells are less affected with the GSH levels showing no significant change compared to control (Fig 7C), and GSSG showing a slight decrease at 1 h NRH exposure, which then becomes significant at 4 h (Fig 7D), but partially reversed at 24 h. As for HEK293T, the de novo biosynthesis of GSH appears to be put on hold in HepG3, but the restoration of (GSSG+GSH) levels is indicative of a need for an effective GSH/GSSG cycling in response to NRH-induced ROS. The little changes in GSH/GSSG, observed in HepG3, support a ROS-associated mechanism following a reductive imbalance in the NADP(H) pool induced by NRH, an imbalance that goes unchallenged in HEK293T.

### NRH exposure alters mitochondrial function in HepG3 but not in HEK293T

**Fig 8:**
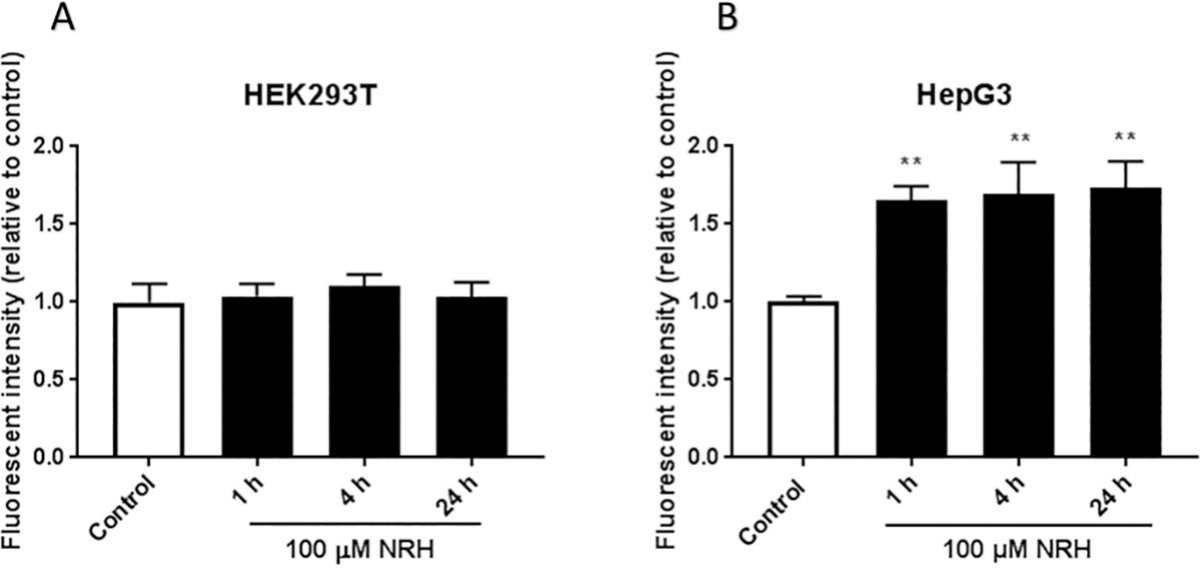
NRH increased mitochondria-derived superoxide formation in HepG3 but not in HEK293T cells. Cellular mitochondrial superoxide production was assessed by measuring the fluorescent intensity of MitoSOX Red in untreated and 100 μM NRH exposed (A) HEK293T and (B) HepG3 cells. Results are presented as mean fluorescence intensity relative to the control ± SEM of three independent experiments. Statistical significance: ** P < 0.01.

As mitochondria are pivotal for life and death decisions and several mitochondrial proteins are ROS-sensitive, mitochondrial oxidative stress could play an important role in determining cell fate [41]. To examine the effect of the cofactor imbalance on mitochondrial oxidative stress, we used MitoSOX to measure the mitochondrial superoxide production in HEK293T and HepG3 cells after exposure with NRH for 1, 4, and 24 h. Live-cell imaging of mitochondria exposed to NRH revealed no significant increase in the superoxide production in HEK293T cells (Fig 8A). As expected, significant increases in superoxide production by 1.66 to 1.73 fold in HepG3 cells between 1 and 24 h of NRH exposure (Fig 8B). These data suggest that NRH rapidly induced mitochondrial superoxide production in the HepG3.

**Fig 9:**
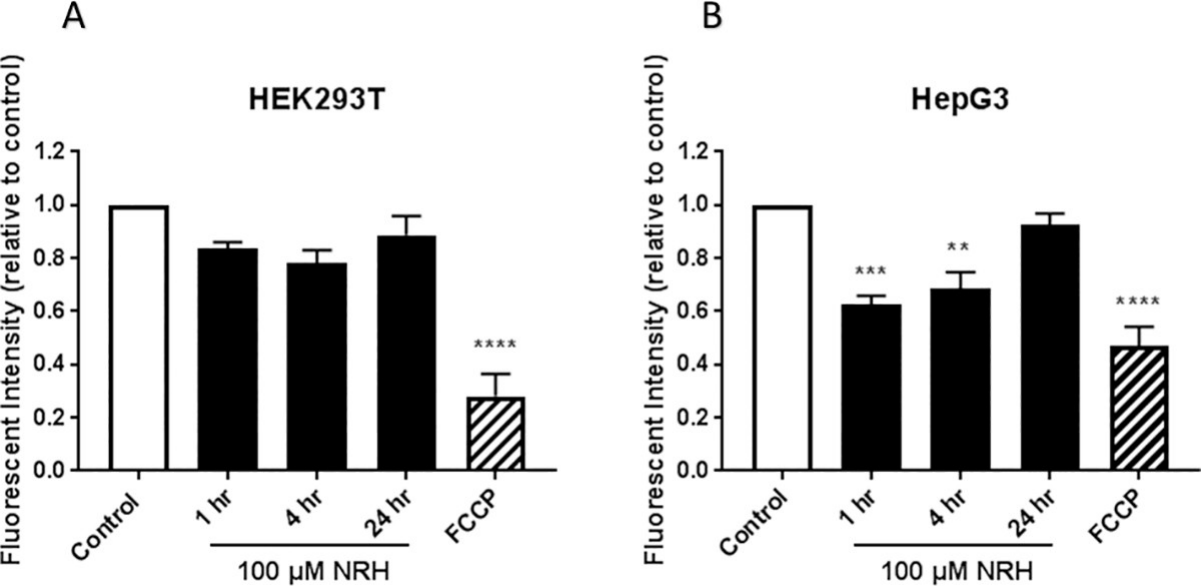
NRH exposure altered mitochondrial membrane potential (ΔΨ_m_) in HepG3 but not in HEK293T cells. Mitochondrial membrane potential was assessed by measuring the mean fluorescent intensity of TMRE (plate assay) in NRH exposed (A) HEK293T and (B) HepG3 cells. FCCP uncouples the membrane potential resulting in loss of fluorescence signal and is thus used as a control. The graph represents the mean fluorescence intensity of three independent experiments normalized for cell number variations (see the Materials and Methods section) ± the SEM. Statistical significance: * P < 0.05, ** P < 0.01, *** P < 0.005 and **** P < 0.001.

Mitochondrial ROS (mtROS) generation is tightly regulated by O_2_ availability, through the concentration and redox state of the electron carrier FADH_2_ enabled by NADH and mitochondrial membrane potential [42, 43]. Since NRH alters the intracellular balance of NAD(P)H (Figs 5 and 6), we examined the effect of NRH on the mitochondrial membrane potential (Δψ_m_) using TMRE. Fluorescence intensity generated by TMRE provides a qualitative measure of the mitochondrial membrane polarization, which is related to the cell’s ability to generate ATP by oxidative phosphorylation. Cells were exposed to 100 μM NRH for 1, 4, and 24 h then stained with TMRE, and the relative fluorescent intensity was measured. Changes in the fluorescence of TMRE were measured and normalized to cell number to capture changes in Δψ_m_. The mitochondria depolarizing agent FCCP resulted in a greater than 50% reduction in Δψ_m_ at 1 h in both cell lines. HEK293T cells exposed to NRH for 1 and 4 h induced a non-significant decrease in Δψ_m_, which then increased back to control after 24 h NRH exposure (Fig 9A). The HepG3 cells showed a 37 ± 3.1% reduction in Δψ_m_ at 1 h NRH exposure. The longer NRH exposure of 24 h further recovers the drop in membrane potential to that of the control (Fig 9B), consistent with the low cytotoxicity observed in HepG3 cells at 24 h exposure (Fig 2B). Together, these data suggest that NRH, likely via NADH oxidation, induced rapid (within 1 h) mitochondrial depolarization in the HepG3 cells but not in HEK293T. This is consistent with the differences in the NAD/NADH ratio observed between HepG3 and HEK293T (Fig 6).

**Fig 10:**
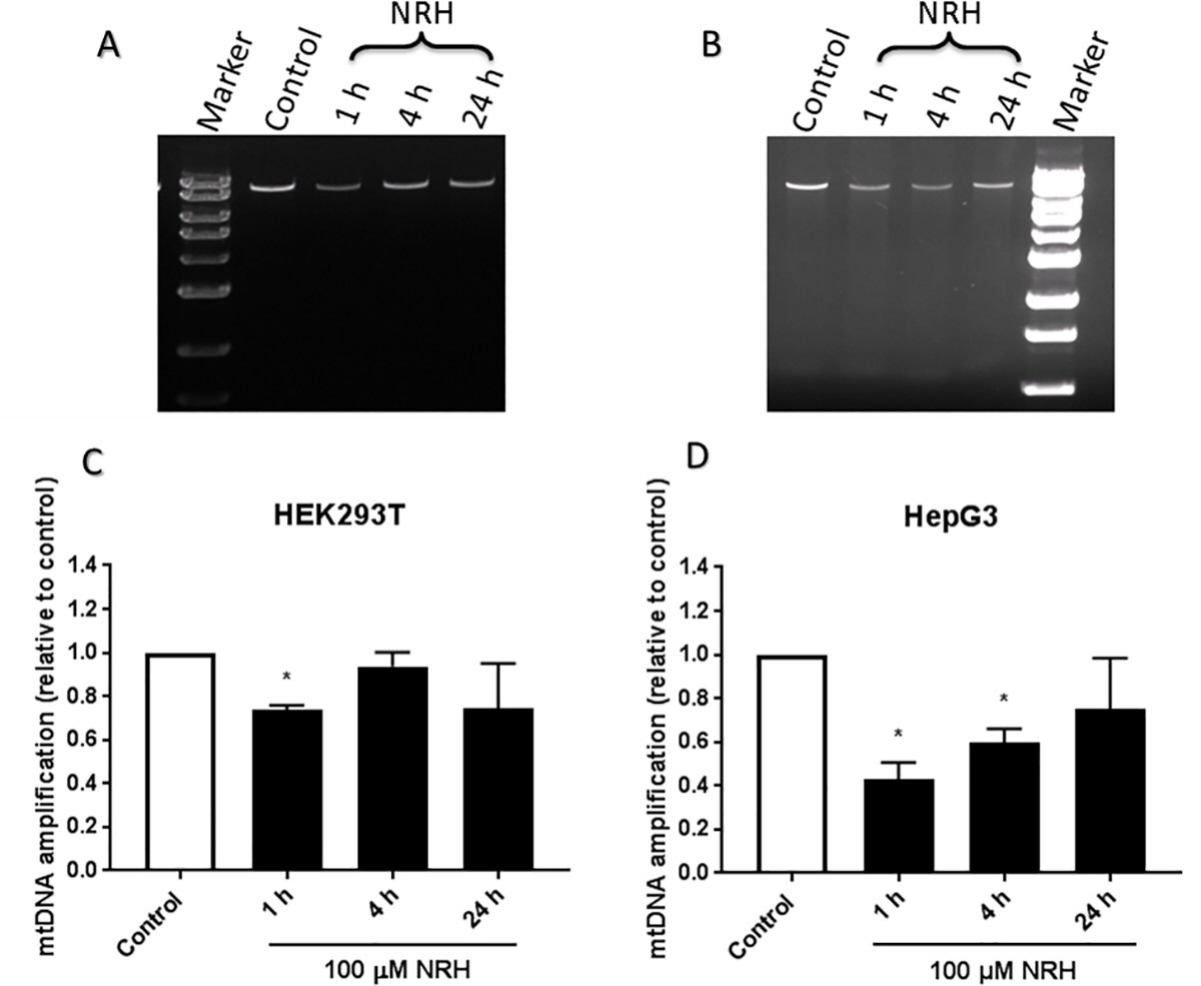
Mitochondrial DNA integrity of NRH-treated HEK293T and HepG3 cells. Electrophoresis and quantitative results from RT-PCR amplified products of mitochondrial DNA measured by QPCR assay (as described in the Materials and Methods section). Representative image of RT-PCR amplified product of mitochondrial DNA on agarose gel, stained with ethidium bromide in (A) HEK293T and (B) HepG3 cells exposed to 100 μM NRH for the indicated times. Quantification of relative amplification levels of mitochondrial DNA (mtDNA) in (C) HEK293T and (D) HepG3 cells from the agarose gel image using Image Lab software. Results are calculated by comparing the mtDNA amplification levels of the exposed samples with undamaged control ± SEM of three independent experiments. Statistical significance: * P < 0.05.

Increased mtROS generation may also lead to mitochondrial DNA (mtDNA) damage that may impair electron transport chain (ETC) function, further enhancing electron leak and ROS production. Thus, we hypothesized that NRH may induce mtDNA damage due to the excessive superoxide production and mitochondrial membrane depolarization. To test this hypothesis, we measured the extent of mtDNA damage using PCR amplification of a long mtDNA region (described in the Material and Methods section). A reduction in the long fragment’s amplification would indicate mtDNA damage as DNA lesions are known to delay or block the progression of DNA polymerase, as previously discussed [44]. At 1 h of NRH exposure, HEK293T cells showed a significant reduction in amplification of the long fragment by 27 ± 2.4%, which then becomes non-significant at 4 and 24 h of NRH exposure (Fig 10A and 10C). In HepG3 cells, NRH exposure at 1 and 4 h significantly reduced the long fragment amplification by 57 ± 7.7% and 40 ± 6.4%, respectively, which then recovered at 24 h of NRH (Fig 10B and 10D). These results show that NRH induces mtDNA damage in both HEK293T and HepG3 cells, with more severe damage observed in HepG3.

### NRH exposure alters mitochondrial respiratory functions in HepG3

**Fig 11:**
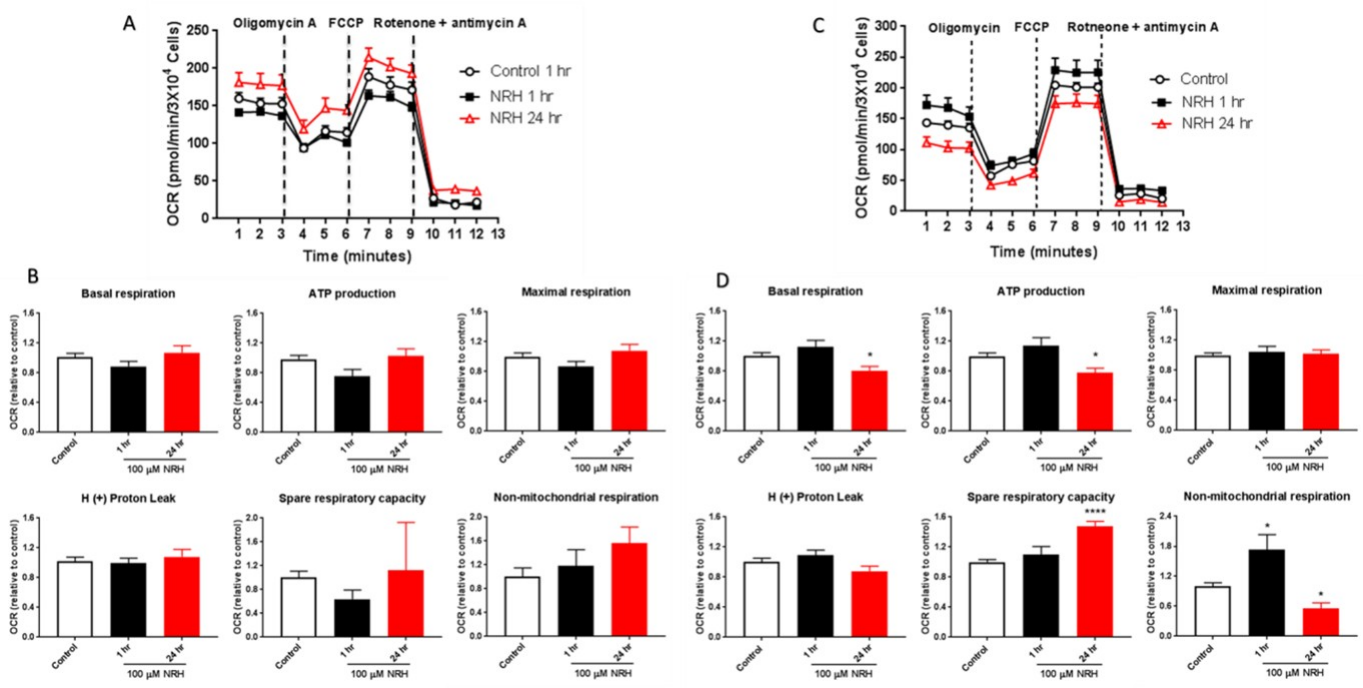
NRH exposure compromised mitochondrial function in HepG3 but not in HEK293T cells. The Seahorse Biosciences XF24 extracellular analyzer was used to measure mitochondrial stress test parameters. Representative OCR curves of untreated (white) and 100 μM NRH exposure for 1 h [39] and 24 h (red) in (A) HEK293T and (C) HepG3 cells. Mitochondrial respiratory function was assessed by adding oligomycin, FCCP and rotenone & antimycin A and individual parameters for basal respiration, ATP production, proton leak, maximal respiration, spare respiratory capacity and non-mitochondrial respiration was measured in (B) HEK293T and (D) HepG3 cells. Results are expressed as mean OCR values relative to control ± the SEM of three independent experiments. Statistical significant *P < 0.05.

With the induction of mtROS and mtDNA, we investigated NRH-induced mitochondrial respiratory dysfunction by Cell Mito Stress Test. Oxygen consumption rate (OCR) values were measured in real-time in cells exposed to NRH for 1 and 24 h at basal levels and following sequential addition of mitochondrial respiration inhibitors: oligomycin, FCCP, and a combination of antimycin A and rotenone (Fig 11). OCR measurements are shown in Fig 11A and 11C, and quantitation of Mito Stress Test parameters are shown in Fig 11B and 11D.

As expected, HEK293T cells showed no significant increase or decrease in all the Mito Stress test parameters when exposed to NRH at 1 and 24 h (Fig 11B). Consistent with the NRH exposure effect observed on metabolic functions in HepG3 cells (Figs 9 and 10), significant alteration of the mitochondrial respiration function was observed in HepG3 cells exposed to NRH. At 1 h NRH exposure, non-mitochondrial respiration increased significantly in HepG3 cells, with no significant change in all other key parameters (Fig 11D). At 24 h NRH exposure, basal respiration, ATP production, and non-mitochondrial respiration decreased significantly by 20 ± 5.6%, 22 ± 6.0%, and 44 ± 10.7%, respectively, compared to control (Fig 11). Spare respiratory capacity also showed a significant increase (47 ± 7.0%), with no change in maximal respiration and H^+^ (Proton) leak (Fig 11D). These observations are consistent with the increased use of O_2_ for non-ATP producing events, such as FAD-dependent oxidation of NADH.

### Mito-TEMPO mitigates mitochondrial specific ROS but not cytotoxicity of NRH in HepG3 cells

**Fig 12:**
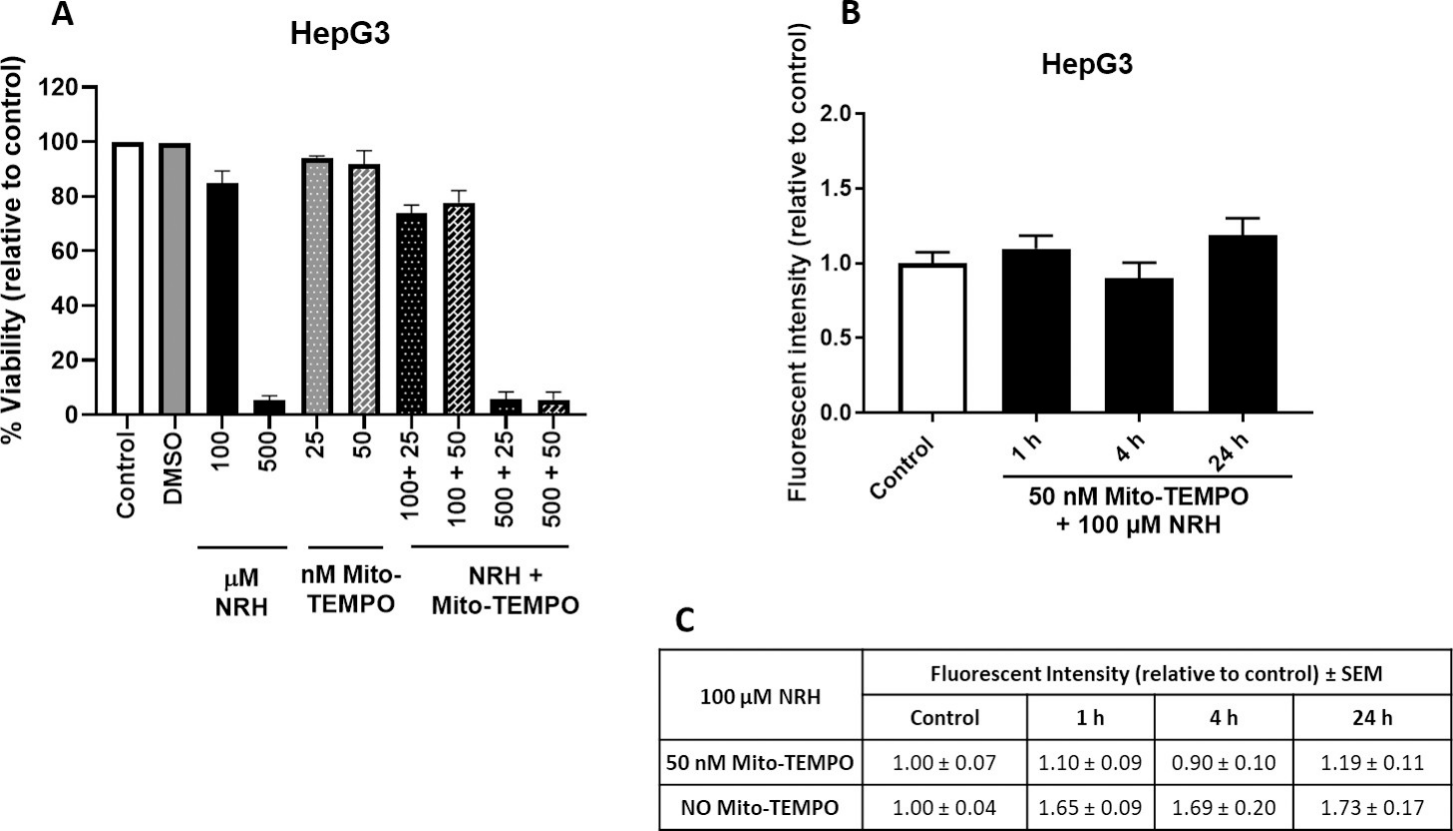
Mito-TEMPO mitigated NRH-induced mitochondrial superoxide production but did not reduced the cytotoxicity. (A) Cell viability was measured by treating HepG3 cells to 100 and 500 μM of NRH alone and in combination with 25 and 50 nM mito-TEMPO for 24 h. After exposure period, NRH was removed and replaced with fresh medium, and cells were allowed to grow for another 48 h before performing CellTiter-Fluor^TM^ Viability assay. Results are expressed as the mean fluorescence intensity to that of the control (% Viability) ± SEM. (B) Cellular mitochondrial superoxide production was assessed by measuring the fluorescent intensity of MitoSOX Red in untreated (control), 100 μM NRH co-exposed 50 nM mito-TEMPO treated HepG3 cells. Results are presented as mean fluorescence intensity relative to the control ± SEM of three biological repeats. (C) Table summarizing the final MitoSOX fluorescence values for NRH exposed HepG3 cells with and without mito-TEMPO treatment ± SEM.

With NRH altering the NAD pool and generating mitochondrial specific ROS, we sought to determine if a NAD pool imbalance or ROS generation was driving cell death. We specifically scavenged the mtROS generated by NRH using mito-TEMPO. Mito-TEMPO combines the antioxidant piperidine nitroxide with the lipophilic cation triphenylphosphonium to create a mitochondria-targeted chemical with effective superoxide scavenging properties [45]. Mito-TEMPO treatment of HepG3 cells did not show any cytotoxicity in HepG3 cells (Fig 12A). We then co-treated HepG3 cells with mito-TEMPO and two different doses of NRH for 24 h and cell viability was measured. Mito-TEMPO could not prevent the cell-specific cytotoxicity in HepG3 cells caused by NRH exposure (Fig 12A).

We verified mito-TEMPO alleviated the NRH-induced mitochondrial specific ROS levels by measuring superoxide generation in the HepG3 cells at 1, 4, and 24 h. Live-cell imaging of mitochondria co-exposed to NRH and mito-TEMPO revealed no significant increase of the mitochondrial superoxide production compared to control in HepG3 cells (Fig 12B). Further comparison of the fluorescent intensity values between with and without mito-TEMPO treatment showed a significant reduction of NRH-induced mitochondrial superoxide production (Fig 12C). These data demonstrate that mitochondrial specific ROS does not drive cytotoxicity in HepG3 cells.

## Discussion

In recent years, studies on NAD^+^ intermediates have demonstrated the beneficial effects of supplementation to maintain tissue NAD levels in aging or age-associated diseases [13, 15]. Most supplementation studies have examined NMN, NR, nicotinic acid, or nicotinamide, but NRH has the highest potency in increasing total NAD concentrations in cells [31, 32]. However, the highly elevated NAD levels that are NRH-induced may have adverse effects, as previously explained by Ziegler and Nikiforov [46]. In cultured cells, NRH exposure resulted in different cellular cytotoxic responses between HEK293T and HepG3 cells (Fig 2). Unlike NRH, NR did not show any cytotoxicity in HepG3 cells (S2 Fig). The cytotoxic effects observed in HepG3 but not in HEK293T after NRH exposure highlights the importance of NRH specific effects in different tissue types. HepG3 is an immortalized cell line more comparable to primary hepatocytes than HepG2 due to its higher metabolic capacity from cytochrome P-450, high albumin, and alpha fetoprotein production [47]. NRH exposure increased BAX and PUMA protein expression levels, inducing mitochondrial-mediated apoptosis (Fig 3D–3F) [48, 49]. These observations suggest that cell-specific responses to NAD increases can cause an imbalance in intra-organelle NAD pools causing cellular dysfunction and eventually inducing cell death.

Consistent with this, we found increased cellular ROS levels in HepG3 but not in HEK293T cells exposed to 100 μM NRH (Fig 4). NAD(P)H levels were also increased in the mitochondria and nucleus of HepG3 cells, supporting the different fates of NAD species within these cell lines (Fig 5). Differential generation of NAD(H) vs. NADP(H) after NRH supplementation was further supported by the MS data (Fig 6) and also by recent work demonstrating NMN adenylyltransferases can generate NADH that is then oxidized to NAD^+^ from NMNH [28]. The dramatic changes in NAD^+^/NADH and NADP^+^/NADPH levels upon NRH supplementation is possible because of a lack of feedback inhibition for NAD^+^ production by NRH. Similarly, the NAD kinase does not appear to have time to be inhibited by NADPH before it generates high NADP^+^ levels from an acute increase of NAD^+^ in HepG3 cells (Fig 6G). Changes in the NADH pool also impact NADPH levels (Fig 6E–6H). NADPH is the only hydride source for the reduction of the GSH pools in mitochondria [50]. The ready conversion of NRH to NAD(P)H in HEK293T supports decreased (GSH+GSSG) levels in this cell line. The more limited and yet trending changes in GSH and GSSG in HepG3 cells indicate an initial reductive environment supplanted by an oxidative environment (Fig 7). NRH exposure and subsequent NADH production cause reductive stress by reducing available metabolic electron acceptors. However, oxidation of the increased NRH and NADH pools uses O_2,_ which increases ROS production as the NRH-driven NAD(P)H pool are equilibrated. Therefore, the cellular redox cycling driven by NRH exposure uses NADP^+^/NADPH and GSH/GSSG redox couples [51, 52]. HepG3 shows cellular dysfunction caused by oxidative mechanisms.

Similar cell-specific NRH-driven cofactor imbalance is observed in mitochondrial oxidative stress, with a significant increase in mitochondrial superoxide production in HepG3 cells but not in HEK293T (Fig 8). If NRH-derived NADH increases (directly or indirectly) mitochondrial NADH, Complex I can be activated and an acute increase in NADH levels may increase electron transfer dysfunction and ROS generation [39]. Similarly and via the nicotinamide nucleotide transferase, an increase in NADH, combined to an increase in NADP^+^, can lead to an increase in mitochondrial NADPH, which is a substrate, if not used for GSH regeneration, for NADPH oxidases via processes that generate superoxide from O_2_ and NADPH [53]. NRH induced significant increases in mitochondrial ROS levels (Fig 8B) and a decrease of Δψ_m_ at 1 and 4 h of exposure in HepG3 cells (Fig 9B). However, this mitochondrial oxidative stress is not primarily responsible for the cytotoxicity associated NRH in HepG3. Mito-TEMPO addition eliminated the generation of mitochondrial superoxide and did not impact the cytotoxicity of NRH. Other radical species are likely produced in the cytoplasm that may contribute to NRH-driven cytotoxicity in HepG3.

Critically, NRH is oxidized to NR by the enzyme NQO2, which uses NRH as a preferred reductive substrate [28, 30]. Increased levels of NQO2 by NRH in HepG3 cells (S3 Fig) could increase cytosolic ROS levels while consuming O_2_; whereas, lower NQO2 levels (S3 Fig) would allow for NRH phosphorylation by adenosine kinase and lead to NADH in the cytosol. Once made available in the mitochondrion, this NADH could then, via nicotinamide nucleotide transhydrogenase (NNT), increase NAD(P)H levels in the mitochondria and a controlled trends of decreased membrane potential (non-statistically significant) in HEK293T (Fig 9A). NQO1 is the homolog of NQO2 that oxidizes NADH and NADPH. NQO1 and NQO2 are both FAD-NAD(P)H dependent reductases, capable of generating ROS. The expression of these FAD-NAD(P)H dependent reductases is shown to be increased in HepG3 cells (S3 Fig). In HEK293T cells, NADH seems to remain unused for a while and does not contribute to ROS producing events. Instead, it appears to be used in glycolysis (straight non-FAD dependent oxidation, not producing ROS) in HEK293T as the mitochondrial respiration measurements indicate (Fig 11A and 11B). Following a period of adaptation, an increase in NAD^+^ provided the mitochondrion with an approach to generate ATP, very different from HepG3, which seems to favor NADH/riboflavin dependent processes and thus, generation of ROS, in particular in the mitochondrion (Fig 8). The more rapid changes in the levels of ROS and Δψ_m_ observed in HepG3 may drive BAX-mediated apoptosis when higher doses of NRH are used. At 100 μM supplementation with NRH, the mitochondrial membrane potential appears to restabilize, though not completely (Fig 9), consistent with the slight cytotoxicity observed in HepG3 cells at this dose (Fig 2B). NRH exposure also induces mtDNA damage in the HepG3 cells, with a significant increase in mtDNA damage at 1 and 4 h of NRH exposure in HepG3 cells (Fig 10B and 10D). The mtDNA damage is mostly repaired at 24 h, but again complete recovery is not observed consistent with the slight cytotoxicity observed.

Consistent with the membrane potential changes and mtDNA damage, NRH exposure altered mitochondrial respiratory function in HepG3 cells but not in HEK293T (Fig 11). Studies have shown that non-mitochondrial OCR typically increases in the presence of oxidative stressors such as ROS [54, 55], which is consistent with the increased levels of superoxide production at 1 h of NRH exposure in the present study. This highlights the deleterious effects on the mitochondria of HepG3 cells exposed to NRH and are regarded as negative indicators of bioenergetic health. A decrease in ATP-linked OCR would indicate low ATP demand, a lack of substrate availability and/or severe damage to oxidative phosphorylation, obstructing the flow of electrons, leading to a lower OCR. A decrease in total ATP levels has also been reported to be inversely proportional to the levels of NAD^+^/NADH by using higher doses of nicotinamide, as nicotinamide is an inhibitor of sirtuins, chief controller of metabolic enzyme activities [56]. It is important to note that bioenergetics dysfunction can counter-intuitively increase the apparent spare respiratory capacity, decreasing bioenergetic capacity.

Altogether, our work demonstrates extreme boosts of the NAD pools by the newly discovered NAD^+^ precursor, NRH, occur in a cell-type specific manner (S1 Graphical abstract). We demonstrated that exposure to high dose NRH induced apoptotic cell death through significant increases in PUMA and BAX protein expression levels. At supplementation levels of 100 μM, cell-specific effects by NRH are mediated through the different metabolic fate of NADH/NADPH in these cells. The differential effects of NRH in HepG3 and HEK293T cells observed highlight the cell-specific importance of NAD pools in cellular homeostasis. Manipulating reductive stress through NRH supplementation expands our understanding of the cellular redox environment and highlights that a rapid boost in NAD can concomitantly increase reducing equivalents [NAD(P)H and GSH], causing detrimental effects within the cell and even in specific organelles. We propose a model that in HepG3 cells, fast conversion of NRH to NR and NADH to NAD^+^ causes an imbalance in the cellular NAD pool, thereby increasing cellular NAD(H)/NADP(H) levels, triggering reductive stress followed by oxidative stress and metabolic stress and cellular dysfunction leading to apoptotic cell death. Mammalian cells responded to NAD precursor supplementation through rapid mechanisms that try to maintain a balanced redox environment and ultimately generate oxidative and reductive damage in different cellular environments. Therefore, to create more beneficial supplements for boosting NAD^+^ biosynthesis, a better understanding of the cellular fate within specific tissues and cells of lower levels of such NAD precursor is needed to reduce its harmful side effects.

## Supporting information

S1 Graphical abstract (TIF)

S1 Fig 1H NMR revealed that 100 μM NRH in DMEM is consumed by cells and removed from the cell culture media. Cell culture media after 1 h cell exposure in the absence of NRH (A) HEK293T and (B) HepG3. (C) NRH stability in DMEM media after 4 h incubation in DMEM in absence of cells. (D) NRH levels remaining in the supernatant of HEK293T culture media after 1 and 4 h, respectively. (E) NRH levels remaining in the supernatant of HepG3 culture media after 1 and 4 h, respectively.
(TIF)

S2 Fig Dihydronicotinamide riboside (NRH) induced cell-specific cytotoxicity in HepG3 but not in HEK293T cells. HEK293T (A) and HepG3 (B) cells were treated with 100 μM of nicotinamide riboside (NR) and NRH for 24 h. Treatment was removed after 24 h, and cells were allowed to grow for another 48 h before measuring fluorescence intensity by CellTiter-Fluor^TM^ Viability assay. Results are expressed as the mean fluorescence intensity relative to control (% Viability) ± the standard error of mean (SEM). Statistical significance: ** P < 0.01.
(TIF)

S3 Fig NRH significantly induced NQO1 and NQO2 but not NOX4 expression levels in HEK293T and HepG3 cells. NQO1, NQO2 and NOX4 protein expression levels in HEK293T and HepG3 cells were assessed using immunoblot after 1, 4 and 24h of NRH exposure. Immunoblotting showing (A) NQO2, (E)NQO1 and (I) NOX4 protein expression levels in HEK293T and (B) NQO2, (F)NQO1 and (J) NOX4 protein expression levels in HepG3 cells were. The graph shows quantified protein expression levels relative to controls for (C) NQO2, (G)NQO1 and (K) NOX4 in HEK293T and (D) NQO2, (H)NQO1 and (L) NOX4 in HepG3 (D) cells. Results are expressed as the average of three biological replicates ± SEM. Statistical significance: * P < 0.05.
(TIF)
