# Supplementary figures and images for: Dihydronicotinamide riboside promotes cell-specific cytotoxicity by tipping the balance between metabolic regulation and oxidative stress
Source: PLoS One. 2020 Nov 9;15(11):e0242174. doi: 10.1371/journal.pone.0242174 (PMC7652347; doi:10.1371/journal.pone.0242174)

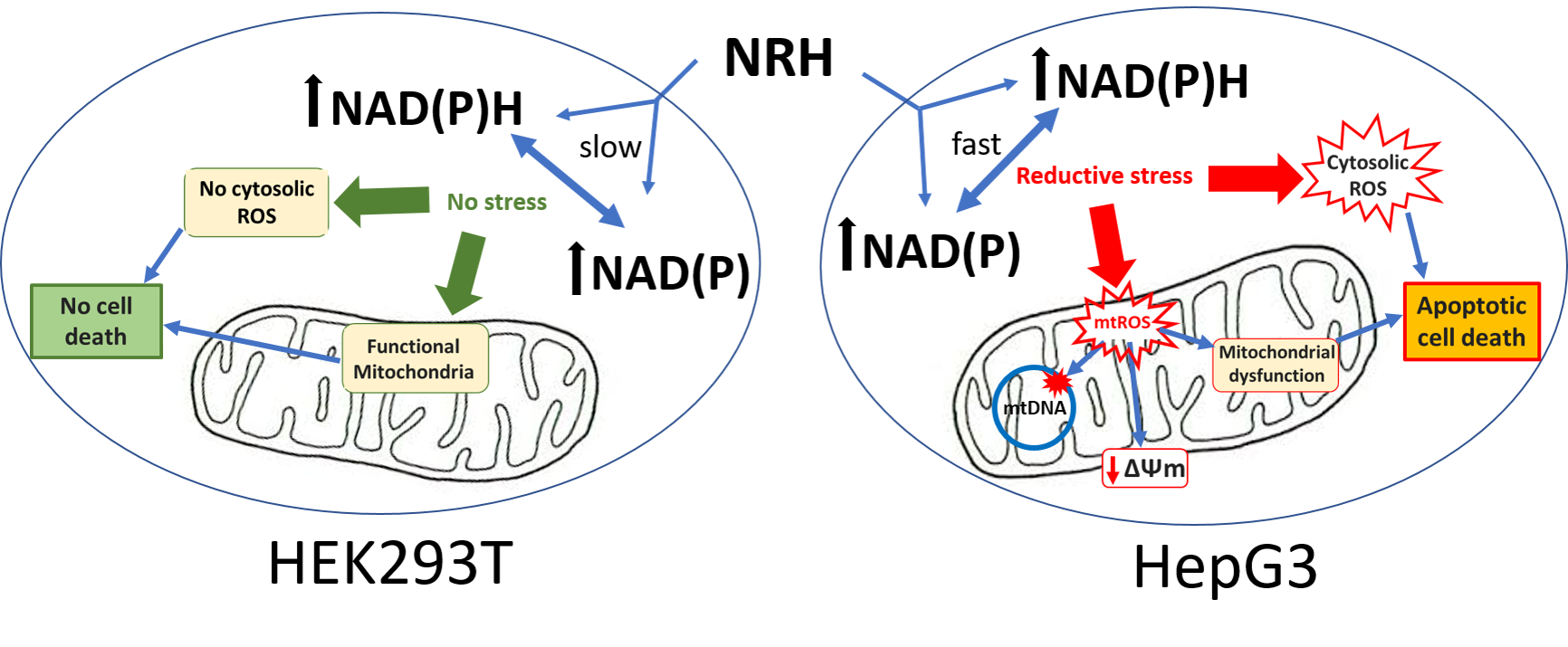

Supplement: S1 Graphical abstract — (TIF) [file pone.0242174.s001.tif]

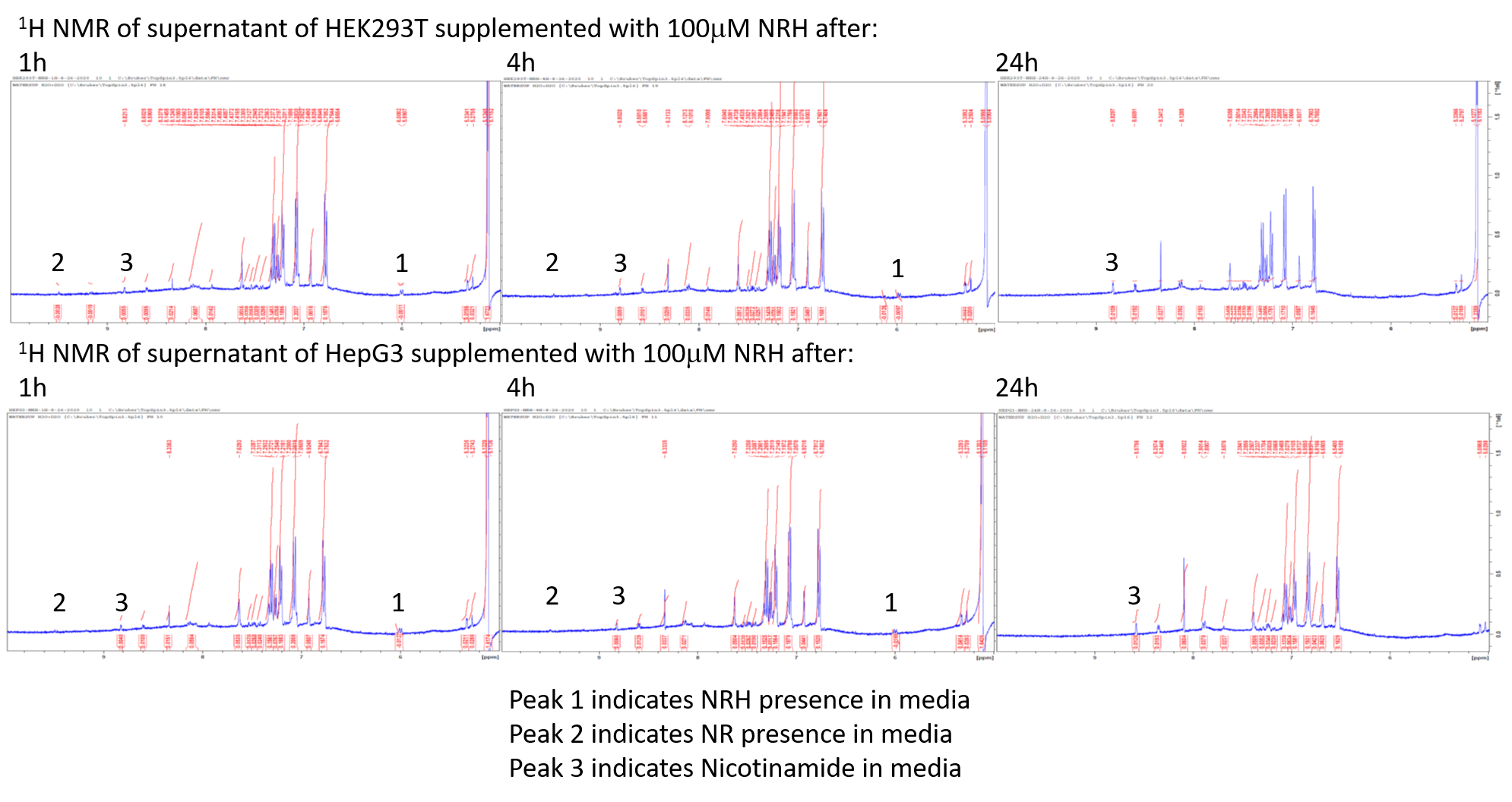

Supplement: S1 Fig — Cell culture media after 1 h cell exposure in the absence of NRH (A) HEK293T and (B) HepG3. (C) NRH stability in DMEM media after 4 h incubation in DMEM in absence of cells. (D) NRH levels remaining in the supernatant of HEK293T culture media after 1 and 4 h, respectively. (E) NRH levels remaining in the supernatant of HepG3 culture media after 1 and 4 h, respectively. (TIF) [file pone.0242174.s002.tif]

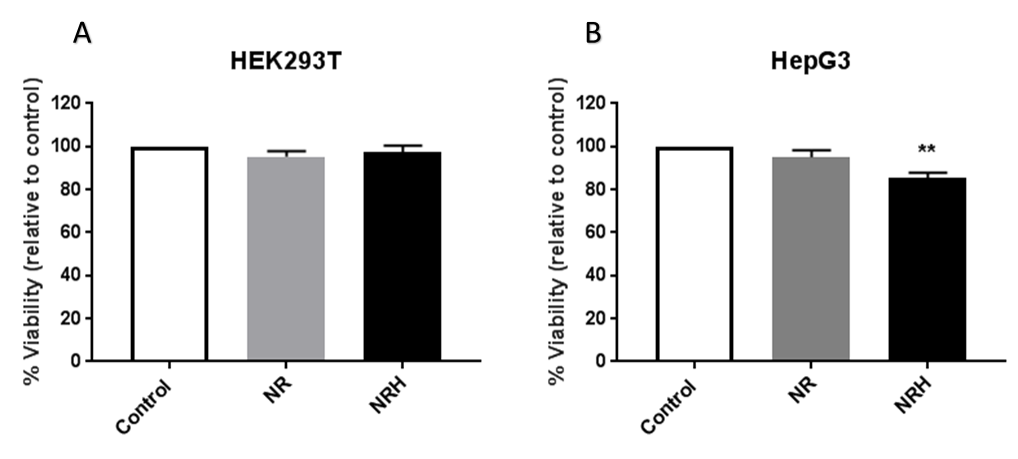

Supplement: S2 Fig — HEK293T (A) and HepG3 (B) cells were treated with 100 μM of nicotinamide riboside (NR) and NRH for 24 h. Treatment was removed after 24 h, and cells were allowed to grow for another 48 h before measuring fluorescence intensity by CellTiter-FluorTM Viability assay. Results are expressed as the mean fluorescence intensity relative to control (% Viability) ± the standard error of mean (SEM). Statistical significance: ** P < 0.01. (TIF) [file pone.0242174.s003.tif]

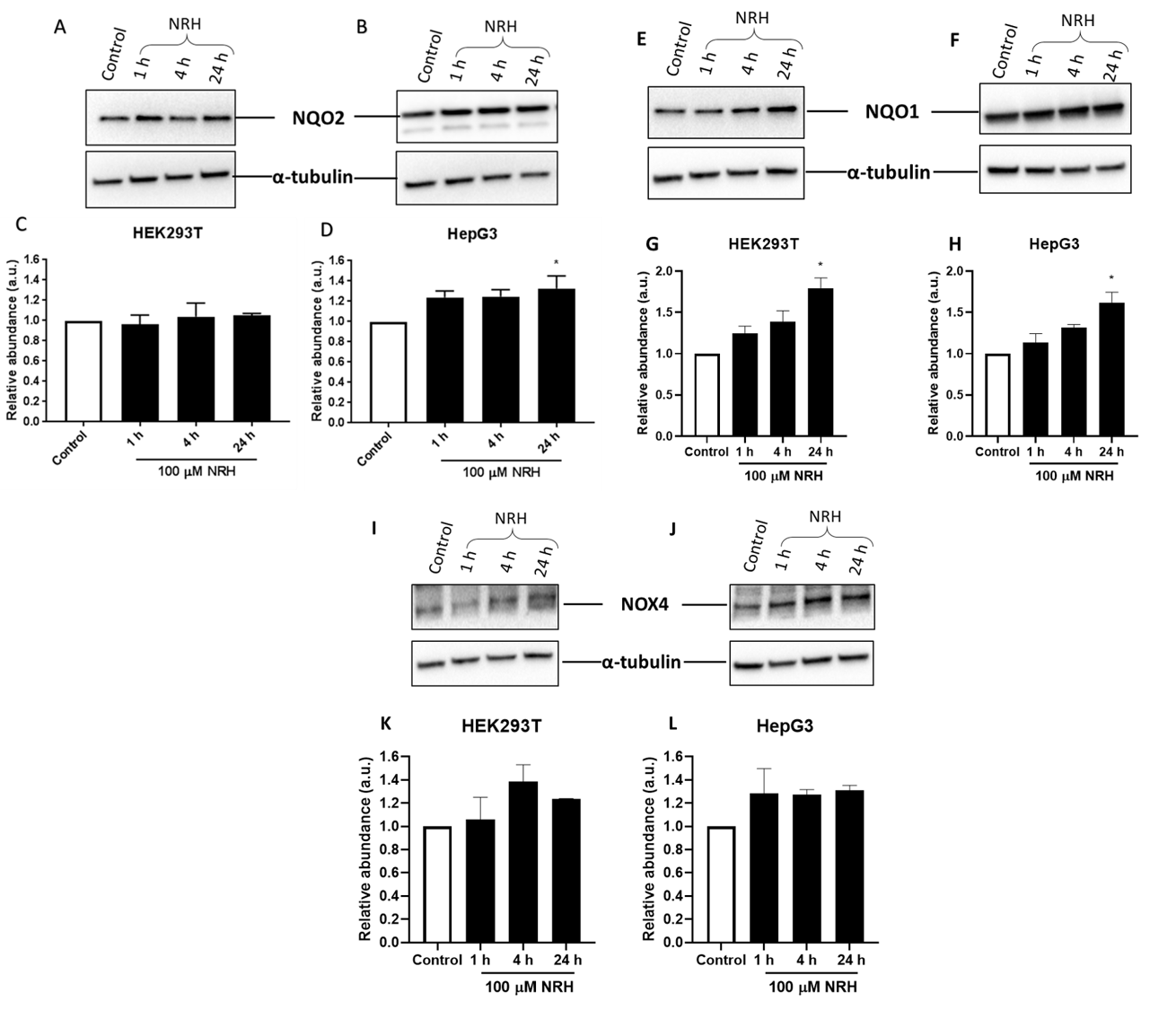

Supplement: S3 Fig — NQO1, NQO2 and NOX4 protein expression levels in HEK293T and HepG3 cells were assessed using immunoblot after 1, 4 and 24h of NRH exposure. Immunoblotting showing (A) NQO2, (E)NQO1 and (I) NOX4 protein expression levels in HEK293T and (B) NQO2, (F)NQO1 and (J) NOX4 protein expression levels in HepG3 cells were. The graph shows quantified protein expression levels relative to controls for (C) NQO2, (G)NQO1 and (K) NOX4 in HEK293T and (D) NQO2, (H)NQO1 and (L) NOX4 in HepG3 (D) cells. Results are expressed as the average of three biological replicates ± SEM. Statistical significance: * P < 0.05. (TIF) [file pone.0242174.s004.tif]
